# Identifiability-Aware, Cost-Aware Triage of Physical Faults and Measurement-Integrity Anomalies in Energy Cyber-Physical Systems

**DOI:** 10.3390/s26175585

**Published:** 2026-09-02

**Authors:** Fuliang Ma, Yuzhen Dang, Yuanming Liu, Tiezhuang Zhou

**Affiliations:** 1 School of Chemical Engineering, Qinghai University, Xining 810016, China; 2 Photovoltaic Industry Technology Branch, Qinghai Huanghe Hydropower Development Co., Ltd., Xining 810007, China

**Keywords:** sensor data integrity, fault diagnosis, cyber-physical systems, measurement anomalies, multivariate process monitoring, decision cost, wind-turbine SCADA

## Abstract

**Highlights:**

**What are the main findings?**

- A strictly alarm-gated, run-disjoint protocol yields conditional physical-feature AUCs of 0.873 on TEP and 0.895 on the wind-farm simulation.
- A separate benign-sensor-fault stress test preserves process-versus-measurement-integrity ranking but forces matched sensor-fault/attack cause AUC to 0.500.

**What are the implications of the main findings?**

- Physical-consistency features provide auditable triage and reduce illustrative episode-level decision cost, but trail a raw-statistics XGBoost benchmark on clean data.
- Observationally equivalent causes require abstention or independent maintenance, sensor-quality, or security evidence rather than a forced telemetry-only label.

**Abstract:**

After an anomaly is detected in an energy cyber-physical system, operators must decide whether to respond as if the process has physically failed or as if the measurements have lost integrity. These causes can require different actions, yet they may produce similar telemetry. We present a detection-gated triage framework that first applies a fixed canonical-variate detector and then uses nine physical-consistency features to distinguish physical process faults from measurement-integrity anomalies. Evaluations on the Tennessee Eastman Process and a wind-farm simulator yielded cause-attribution AUCs of 0.873 and 0.895 and end-to-end balanced accuracies of 0.805 and 0.792, respectively. Under the specified response-cost matrices, the policy reduced expected misattribution cost by 18.4% and 28.9% relative to blanket responses. Tests on real datasets show that ranking can transfer, but operating thresholds require plant-specific calibration. Exact matched sensor-fault/attack pairs give chance-level discrimination, demonstrating a fundamental boundary: measurement-only data cannot identify different causes that generate the same observations. The framework is therefore intended as an auditable triage aid, with ambiguous or out-of-distribution cases routed to review rather than treated as confirmed cyber attribution.

## 1. Introduction

Sensor-rich energy cyber-physical systems (CPSs) depend on transmitted measurements to detect abnormal operation and select a response. A modern wind farm, for example, streams supervisory control and data acquisition (SCADA) signals describing wind speed, power, rotor and generator speed, drivetrain temperature, pitch, and vibration. Multivariate statistical process monitoring (MSPM) methods such as principal component analysis (PCA), dynamic PCA, kernel PCA, and canonical-variate analysis (CVA) can detect departures from the learned normal regime [1,2,3,4]. The resulting alarm establishes that the received sensor vector is abnormal; it does not establish why.

This distinction matters because a physical process fault and corruption of a transmitted sensor channel can trigger the same control-limit violation but require different actions. A genuine gearbox-bearing temperature rise calls for maintenance. A false-data injection that creates the same apparent rise calls for channel isolation, incident response, and a resilient estimate. Routing an alarm to the wrong barrier can therefore leave either physical degradation or a measurement-integrity anomaly untreated. Safety and security must increasingly be assessed together [5,6], yet their joint treatment at the post-alarm diagnostic stage remains limited.

Existing work approaches this problem from two sides. Process-monitoring and wind-turbine condition-monitoring methods detect and isolate physical faults [7,8,9], but generally do not include an adversarial explanation for the same sensor signature. CPS-security methods detect false-data injection and construct resilient estimators [10,11,12,13], but commonly evaluate attacks without placing physical faults in the same confusion matrix. Power-system studies have directly compared attacks and natural events [14,15,16,17,18]; however, many rely on system-specific logs or signatures and do not connect the attribution decision to a sensor-monitoring detectability boundary or a bounded-error estimator.

**Figure 1:**
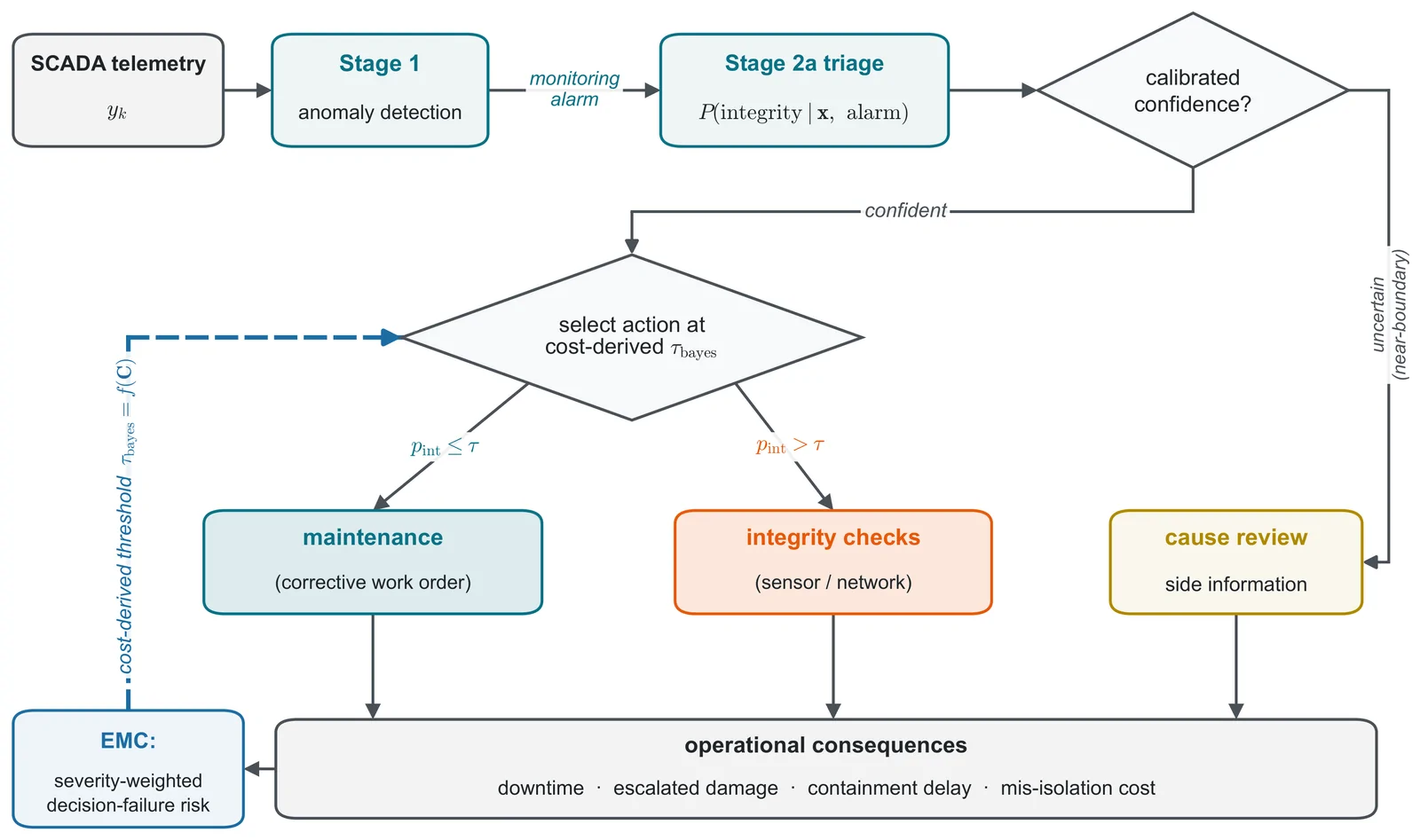
Cost-aware sensor-data attribution and response selection. A matched-false-alarm monitor detects an abnormal measurement window (Stage 1), and a physical-consistency model first triages the alarm as a physical process fault or a measurement-integrity anomaly (Stage 2a). Lower-level sensor-fault/attack decisions are made only when supported; otherwise the episode is escalated for review. Expected misattribution cost (EMC) determines the operating threshold and quantifies the consequence of routing an alarm to the wrong response.

We address this gap as a sensor-data triage problem. Stage 1 detects an abnormal received measurement vector at a matched false-alarm rate. Stage 2 then extracts nine physical-consistency features from the alarm window. Under the evaluated supervisory-telemetry model, physical process faults may propagate through plant coupling, whereas a measurement-layer anomaly may remain concentrated on the affected telemetry channels. This contrast is a conditional modeling tendency, not a guaranteed separator: a localized fault may remain concentrated, and corruption that enters a controller feedback path or is coordinated across channels may become spatially diffuse. The features quantify residual-energy concentration, innovation and noise structure, and the spatial extent of cross-correlation change. An innovation-based defense addresses sustained attacks that suppress the static residual. A reject option is retained as an exploratory deployment mechanism rather than a validated performance claim. Figure 1 summarizes the resulting sensing and decision loop.

The framework is not presented as the most accurate clean-data classifier. A random forest trained on raw per-channel statistics and an XGBoost baseline [19] both achieve higher clean-data discrimination. The physical-consistency layer is instead evaluated for properties important to a deployed sensing system: signal-level auditability, compact features, and traceability to sensor-level evidence. Calibration and abstention are reported separately because the available number of independent runs is limited.

The theoretical results are stated at the level they support. For the PCA squared prediction error (SPE) channel, the residual-subspace dimension sets a support-size transition at which zero-residual attacks exist. The deployed $T^{2}\;\vee \;\mathrm{SPE}$ monitor closes that exact transition, so the result characterizes a monitoring channel rather than proving attribution identifiability. An observational-equivalence result additionally formalizes when benign sensor faults and attacks are not causally identifiable from the received measurements. Further results establish an innovation signature and a uniformly ultimately bounded resilient estimate under stated assumptions. Attribution and EMC performance remain empirical results conditioned on the physical-consistency model.

We test the system on a physics-based wind-farm simulator and the Tennessee Eastman Process (TEP), then examine transfer on real sensor records. The CARE wind-turbine experiment provides the most important boundary result: Stage 2 ranking transfers to real fault telemetry, but the decision threshold learned from synthetic contrasts does not. A label-free threshold anchored on plant-normal data restores most of the separation at a cost in attack recall. The evaluation therefore supports plant-anchored calibration, not out-of-the-box real-wind attribution. We therefore extend the binary benchmark with an independent synthetic benign sensor-fault class. Six distinctive sensor-fault mechanisms probe three-class coverage, while exact matched pairs assign different causal labels to identical received trajectories. The latter are a negative control: a measurement-only sensor-fault/attack classifier must remain at chance on those pairs. We consequently use “measurement integrity anomaly” for the hierarchical output and reserve “attack-consistent” for the simulated attack label. Cyber causation requires evidence beyond the classifier and the measurement-layer threat model of Section 3.1.

The main contributions are as follows.

1. We formulate post-alarm fault–anomaly triage as a sensor-data integrity problem and introduce a two-stage pipeline based on nine auditable physical-consistency features. The pipeline includes channel-concentration descriptors that may guide inspection and an innovation defense, but the present study does not validate exact channel or subsystem localization (Section 3.5).
2. We characterize the monitoring limits through an SPE-only covert magnitude budget and support-size transition, an innovation result, and an attack-magnitude-independent estimation-error bound. We further prove an observational-equivalence limit for measurement-only cause attribution. Each result is separated from the empirical attribution claims it does not prove (Section 4).
3. We evaluate the complete detection-gated pipeline at matched false-alarm rates, with a declared commissioning segment, five complete-run outer folds, nested calibration, run-bootstrap intervals, a paired-background check, and a learned baseline. The real-data study distinguishes ranking transfer, threshold transfer, and fully real cross-domain evidence rather than treating them as equivalent. A grouped three-class stress test adds synthetic benign sensor faults, exact causal-relabel controls, and selective review (Section 5 and Section 5.6).
4. We connect run-level triage to operational response through exact episode-level EMC. Missed Stage-1 episodes are charged explicitly, and calibration and rejection are presented as exploratory analyses rather than as hidden improvements to the primary result (Section 6).

The academic contribution is therefore not a claim that one classifier universally separates faults from attacks. It is an identifiability-aware formulation and evaluation protocol for the decision that follows anomaly detection: the protocol separates detection from attribution through a fixed gate, treats exact observational equivalence as a required negative control, and maps uncertainty to asymmetric response costs and an explicit review action. This structure exposes both when telemetry supports a defensible triage decision and when the cause is not identifiable from measurements alone.

The remainder of the paper reviews related work, defines the measurement and threat models, presents the method and detectability analysis, reports simulation and real-data results, evaluates risk-aware decisions, and closes with limitations and deployment requirements.

## 2. Related Work

### 2.1. Multivariate Statistical Process Monitoring and Fault Detection

Multivariate statistical process monitoring (MSPM) is the dominant paradigm for detecting abnormal behavior in industrial processes from correlated sensor streams. Principal component analysis and its dynamic and kernel extensions trace to the disturbance-detection and canonical-variate-analysis formulations of [1,2], consolidated in the standard reviews of [3,4]. Canonical-variate dissimilarity for incipient faults [20] and kernel-based nonlinear monitoring [21] extend this family toward finer-grained diagnosis. Detectability-aware controller screening [22] connects process monitoring to the residual-subspace arguments used later in our detectability proposition. The recent data-driven-monitoring review of [7] confirms the field’s maturity, while the TEP comparison of [23] establishes PCA-family detectors as standard comparative baselines. These foundations motivate our Stage-1 detector bank (PCA, DPCA, CVA, and KPCA). The PCA-charting line remains under active development in the reliability literature: covariate-regulated principal components extend $T^{2}$/SPE charting to time-varying operating regimes [24]. This is directly relevant to the noise-drift limitation we disclose in Section 7: an adaptive-limit scheme of that family is the natural mitigation for the FAR inflation our stationarity assumption incurs. Across this entire body of work, however, a control-limit violation is interpreted only as “a fault,” because no adversarial alternative is ever entertained in the evaluation protocol.

### 2.2. Cyber-Physical System Attack Modeling and Detection

A parallel stream of the literature formalizes sensor and actuator attacks on cyber-physical systems and derives detectors for them. False-data-injection attacks that evade classical bad-data detection were introduced by [10], with replay attacks analyzed by [25]. Control-theoretic attack detection and identification is developed by [11], and secure estimation under sparse adversarial corruption is developed by [12]. Ref. [13] frames attacks in terms of the resources available to a resource-limited adversary, formalizing the covert and stealthy attack classes we adopt in our threat model. The physics-based detection survey of [26] maps the principal sensor-channel threat models and defenses. This literature motivates the measurement-layer scope adopted here. A related thread studies *adaptive* adversaries that know the defender’s mechanism and optimize against it directly [13]. The resource-limited attack budget formalized there is precisely the worst case such an adversary exploits. Probabilistic cost–benefit models of attack-path selection [27] supply the attacker-side expected-loss calculus whose defender-side mirror is the consequence matrix of Section 6. The reliability literature has recently taken up the same thread on its own terms. False-information injections on demand-response programs are detected online with convolutional networks whose accuracy degrades most on the variable-rate, adaptive injections that matter most [28]. Federated unsupervised detectors flag protocol-level attacks across control center and substations without manually preset thresholds [29]. Multi-tier frameworks detect bidirectional false-data injections explicitly framed as reliability enhancement [30]. In every case, however (control-theoretic and reliability-native alike), the evaluation protocol assumes the anomaly is known a priori to be an attack. None of these works place a physical-fault scenario in the same confusion matrix, so their reported detection rates cannot be read as attribution accuracy, a gap our Stage-2 discriminator is designed to close.

Recent *Sensors* studies help separate adjacent parts of the resilience chain. Hard/soft physical failures and cyber-attacks can be modeled jointly at the closed-loop system level [31]; controller cyber-attacks can be detected and isolated with observer-based residuals [32]; and sensor-fault diagnosis surveys organize mechanisms by error form and temporal behavior [33]. Security-control surveys further distinguish detection, diagnosis, resilient estimation/control, and recovery [34]. The present study occupies the post-detection triage step: it does not replace an observer bank for exact fault isolation or a resilient controller, and its evaluated measurement anomaly is located on the supervisory telemetry path rather than inside the controller feedback loop.

### 2.3. Resilient Estimation and Secure Control

A related thread designs estimators and controllers that remain bounded-error under sensor corruption rather than only flagging it. Event-triggered observers robust to sparse sensor noise and attacks [35], joint attack detection and secure estimation [36], and sliding-mode-observer-based attack isolation [37] establish representative bounded-error and attack-isolation constructions. This literature supplies the theoretical vocabulary (bounded estimation error, residual subspace, detectability margin, covert attack headroom). We repurpose it for a *discrimination* guarantee (is the anomaly a fault or an attack) rather than only a robustness guarantee (keep the estimate bounded regardless of cause).

### 2.4. Wind-Farm SCADA Condition Monitoring

Wind-turbine condition monitoring from SCADA data is itself a mature sub-field. Normal-behavior modeling of turbine subsystems was established by [38]. Recent reviews [8,9] chart the evolution from signal-processing to deep learning-based turbine diagnostics. The bearing-specific interpretable model of  [39] shows that component-level fault detection from SCADA telemetry is well developed and that the wind-speed/active-power coupling is already established as a key physical-consistency anchor for detecting degradation. Semi-supervised multivariate detectors now reach F1 ≈0.99 on real turbine SCADA streams [40], yet emit an undifferentiated “anomaly” verdict with no attribution between physical degradation and data-integrity manipulation. The attack surface is documented: wind-farm control networks are penetrable and horizontally traversable at farm scale [41], and the exposure is not hypothetical. The February 2022 KA-SAT satellite-network attack [42,43] disrupted remote monitoring and control links for 5800 Enercon turbines, a fleet exceeding 10GW, and 2021–2022 ransomware intrusions at Vestas, Nordex, and Deutsche Windtechnik cut remote access to some 2000 turbines [44]. These incidents played out at the IT/communications layer and released no labeled plant telemetry. Still, no condition-monitoring work above considers an adversarial alternative to a flagged turbine anomaly, despite SCADA telemetry traversing the measurement-layer attack surface of the CPS-security literature.

### 2.5. The Fault-Versus-Attack Attribution Gap

A cluster of work poses the discrimination question directly, concentrated in power-system protection rather than process or wind-farm monitoring. The earliest and, methodologically, the closest antecedent is the MSU/ORNL line of Hink et al. [45] and Pan et al. [14]. That line trained classifiers to separate real attacks from natural events and normal operation on a hardware-in-the-loop power-system testbed a decade ago: a genuine three-way attribution formulation, not merely the dataset we reuse in Section 5.6. Its evidence is system-specific, though: the classifiers mine synchrophasor measurements with relay and system logs, so the approach does not transfer to plants exposing only process telemetry, and it carries no detectability theory. More recently, Refs. [15,16] discriminate cyber intrusions from physical faults in intelligent-electronic-device and power-electronic settings. The systems of [17,46] correlate cyber and physical event streams to separate substation faults from attacks in real time. The analysis of [18] formalizes the distinguishability of anomalies as physical faults or actuation-layer cyberattacks. In the nuclear domain, the dynamic-probabilistic-risk-assessment line of Smidts and co-workers reasons about whether an abnormal plant event is component-fault-induced or cyber-attack-induced. Most recently, it couples DPRA with game-theoretic response selection [47], though on plant-model and PRA structure rather than measurement-only telemetry. The attack-identification framework of [11] remains the closest control-theoretic antecedent, identifying an assumed attack’s support rather than first deciding whether an anomaly is an attack at all. Closest in spirit among very recent work, Xue et al. [48] propose a dual-detector framework whose two residual subspaces are proved to separate plant faults from integrity attacks in a generic closed-loop control system, pursuing both the detect-then-discriminate structure and a detectability condition; it operates on generic control-loop residuals and carries no consequence-weighted decision layer, calibrated-UQ/abstention, or wind-farm/TEP validation. Complementary to the measurement-only setting, Ref. [49] differentiates faults from cyberattacks in energy systems by fusing cyberspace logs with physical measurements, reinforcing rather than displacing the telemetry-only differentiator we pursue. Relative to this cluster, our increment is (i) the measurement-only setting (no relay or system logs), (ii) the control-theoretic detectability boundary, and (iii) the consequence-weighted decision layer. Machine learning anomaly detectors that could in principle be repurposed for this task are commonly trained and evaluated as one-class or two-class normal-versus-anomaly problems  [40,50], not as three-way fault/attack/normal attribution tasks. They do not supply a control-theoretic detectability boundary alongside the classifier. Our work targets this seam with a wind-farm SCADA case and a Tennessee-Eastman-Process cross-domain structural-generalization benchmark detect-then-discriminate framework. It is paired with a resilient innovation-based estimator and a formal detectability boundary tied to the residual-subspace dimension. The framework attributes the *cause* of a flagged anomaly rather than only reporting its presence. Table 1 summarizes this positioning against the closest antecedents along the capabilities a deployment needs.

### 2.6. Reliability and Safety-Consequence Perspectives

The perspective this paper adopts (that the quantity worth minimizing is not classification error but the *expected consequence* of a wrong operational decision) is the native language of reliability and system-safety engineering. Risk-informed decision-making frames protective actions by the product of event probability and consequence. The defense-in-depth principle holds that no single protection layer should be relied upon absolutely. Both were formalized in the reliability literature decades ago [51] and remain the reference frame for safety-critical operations. Recent work in this tradition treats the cyber-physical system as the unit of analysis: reliability and performance of coupled cyber-physical systems [52], and dynamic cross-layer security-risk assessment for cyber-physical power systems [53]. These examine how a security event propagates into an availability or safety consequence rather than stopping at detection. In parallel, uncertainty quantification has become central to energy-asset reliability, from global sensitivity and structural UQ for wind-turbine blade reliability [54] to response estimation under environmental uncertainty [55]. This literature treats quantified uncertainty, not a point prediction, as part of the deliverable. Recent uncertainty-informed fault diagnosis [50,56] and confidence-calibrated machinery diagnostics [57] bring the same principle closer to the present decision setting. Our classifier-probability calibration (isotonic, with ECE reported) is the decision-layer analogue of that discipline, not the same technique. Our contribution sits inside this frame: we supply the missing decision layer between a condition-monitoring alarm and the maintenance-versus-security response. We quantify the reliability consequence of getting that decision wrong through an expected-misattribution-cost objective and deliver calibrated confidence with an explicit abstention option. We characterize the safeguard’s failure boundary so that the residual risk is named and mitigable by defense in depth rather than unquantified.

## 3. Materials and Methods

### 3.1. Problem Formulation and Threat Model

#### Measurements and Monitoring Model

Consider a plant instrumented with *n* sensor channels. Let $x_{k}\in R^{n}$ denote the true measurement vector at discrete time *k* and $y_{k}\in R^{n}$ the *received* (telemetered) vector that the central monitor actually observes. To make the control-loop placement explicit, let $y_{k}^{\mathrm{ctrl}}$ denote the value available to the plant controller and $y_{k}^{\sup}$ the value delivered to the supervisory monitor. The evaluated benchmark model uses $y_{k}^{\mathrm{ctrl}}=x_{k}+v_{k}$ and $y_{k}\equiv y_{k}^{\sup}=y_{k}^{\mathrm{ctrl}}+E_{S}a_{k}$; thus, the added measurement-integrity anomaly is on the supervisory telemetry path after the feedback branch. The controller is not driven by $y_{k}^{\sup}$. From a fault-free training record the monitor estimates the mean μ and standard-deviation matrix D=diag(σ) and forms the standardized measurement

(1) $$z_{k}=D^{-1}\left(y_{k}-\mu \right).$$

A PCA model retains *ℓ* principal components in a loading matrix $P\in R^{n\times \ell }$ with $P^{⊤}P=I_{\ell }$, chosen by a cumulative-variance criterion (*ℓ* is reserved throughout for the retained PCA order; *k* indexes discrete time). Writing the principal and residual projectors $\Pi =PP^{⊤}$ and $\Pi^{⊥}=I_{n}-PP^{⊤}$, the two standard monitoring statistics are Hotelling’s $T^{2}$ and the squared prediction error (SPE, or *Q*-statistic):

(2) $$T_{k}^{2}=z_{k}^{⊤}P\Lambda^{-1}P^{⊤}z_{k},\;{\mathrm{SPE}}_{k}={∥\Pi^{⊥}z_{k}∥}^{2},$$

with $\Lambda =\mathrm{diag}(\lambda_{1},\ldots ,\lambda_{\ell })$ being the retained eigenvalues. Control limits $T_{\alpha }^{2}$ and $Q_{\alpha }$ are set at the (1−α) empirical quantile of the fault-free calibration set. An anomaly is declared when a statistic exceeds its limit for a debounce count of consecutive samples. The *residual-subspace dimension* d=n−ℓ is central to the theory of Section 4.

### 3.2. Anomaly Regimes

The received stream is assigned to one of four declared regimes:

- **Healthy:** $y_{k}=x_{k}$ and $x_{k}$ follows the normal-operation distribution.
- **Physical fault:** An internal degradation or component fault perturbs the plant *state*. The perturbation propagates through the process coupling (the plant Jacobian) and appears, correlated, across a broad set of physically coupled channels of $x_{k}$; still, $y_{k}=x_{k}$ (the sensors report truthfully).
- **Benign sensor fault:** The plant state may be healthy, but an instrument, acquisition buffer, or communication component corrupts one or more reported channels without malicious intent.
- **Attack-consistent sensor anomaly:** The plant is physically healthy ($x_{k}$ normal) but an adversary corrupts the telemetered values on a subset of channels.

### 3.3. Threat Model

We restrict attention to *measurement-layer* attacks. Let S⊆{1,…,n} with |S|=s be the *attack support* (the corrupted channels) and $E_{S}\in R^{n\times s}$ the corresponding column selector. The adversary injects an additive corruption $a_{k}\in R^{s}$:

(3) $$y_{k}^{\sup}=y_{k}^{\mathrm{ctrl}}+E_{S}\;a_{k},\;\mathrm{equivalently}\;z_{k}^{\mathrm{atk}}=z_{k}+D^{-1}E_{S}\;a_{k}.$$

We consider four attack families used throughout: additive *bias*, *replay* of previously recorded healthy telemetry, *denial-of-service* (freeze/zero on S), and a model-aware *stealthy* false-data-injection attack that shapes $a_{k}$ to minimize the induced monitoring statistics (Section 4). Following [13], the stealthy adversary is assumed to know the monitor’s model (μ,D,P,ℓ) and the control limits; the bias/replay/DoS adversaries require progressively less knowledge.

**Assumption** **1** 
(Scope)**.** *Attacks corrupt sensor telemetry only, per *(3)*. In the evaluated scope the corruption is introduced after the controller feedback branch and before supervisory monitoring. Corruption of $y_{k}^{\mathrm{ctrl}}$ is not evaluated; if a corrupted sensor value drives the controller, closed-loop dynamics may spread the anomaly across the plant and invalidate the assumed spatial contrast. Actuator-channel manipulation (e.g., pitch-command or manipulated-variable tampering), control-law modification, and coordinated closed-loop attacks that co-design actuation and sensing are outside the scope of this work. Physical faults are modeled as state perturbations expressed through process coupling, whereas attacks act directly on a selected telemetry support. This is a modeling distinction, not a universal physical law: localized equipment faults and benign sensor faults can violate it. The monitoring model further assumes a complete, synchronously-sampled measurement vector $y_{k}$ at each step: missing samples, asynchronous channel timestamps, and communication dropout are not modeled. This idealization holds for the simulator and for the resampled 10-min SCADA records used here, but it is a substantive deployment assumption. The CARE result of Section 5.6 shows that an operating point trained on idealized telemetry must be checked and, where necessary, recalibrated on plant data.*

The simulated attack class instantiates cyber-attacks under this threat model, but an observable output is not itself a causal certificate. Drift, calibration loss, stuck-at behavior, stale buffering, quantization, excess noise, and intermittent spikes can all produce channel-confined measurement signatures. The identifiability stress test therefore adds *benign sensor fault* as an independent declared class. Some sensor-fault mechanisms are distinctive, whereas others are deliberately made observationally identical to an attack. Within this paper, “process fault” means a physical process or equipment fault, “measurement-integrity anomaly” pools sensor faults and attacks at the safe hierarchical decision level, and “attack-consistent” denotes the simulated attack label without claiming cyber causation.

### 3.4. Problem Statement

Given the received stream $\left\{y_{k}\right\}$, we require a monitor that

1. *Detects* an anomaly (Stage 1); 
2. Upon detection, *triages* it hierarchically as a process fault or a measurement-integrity anomaly and reports channel-concentration descriptors that may guide inspection, without claiming exact channel, subsystem, or support localization (Stage 2a); 
3. Attempts the lower-level {sensorfault,attack,review} decision only when the evidence supports it (Stage 2c).

This must account for the operating constraint that a Stage-1 alarm produced by a process fault, sensor fault, or attack can be indistinguishable at the detection layer. The primary binary experiment retains the historical process-fault/attack-consistent-anomaly comparison for continuity. The added hierarchical experiment asks the safer question first and uses exact matched sensor-fault/attack pairs to test whether the cause branch contains information at all. Section 4 characterizes detectability and causal-identifiability limits, while Section 3.5 gives the empirical construction conditioned on the physical-consistency assumption.

### 3.5. The Detect-Then-Discriminate Framework

**Figure 2:**
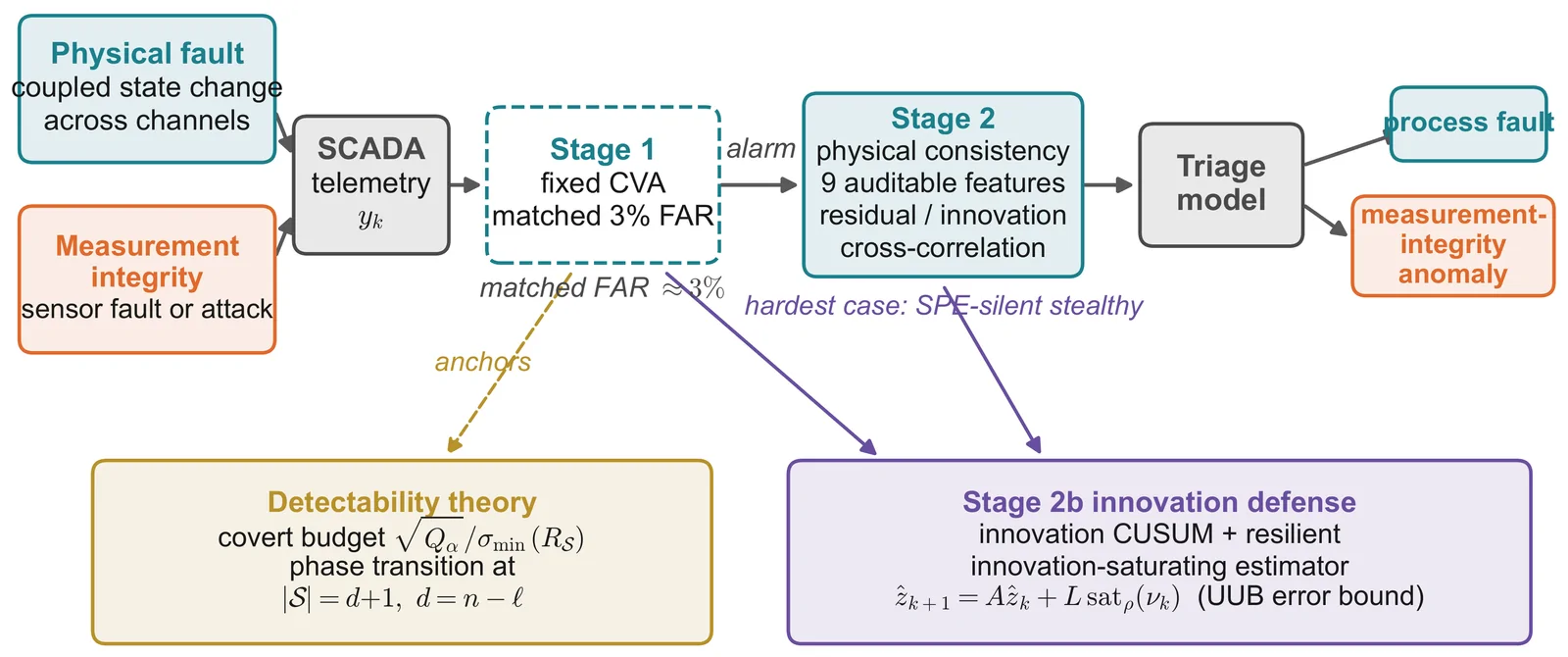
The two-stage detect-then-discriminate framework. Stage 1 (calibrated fixed CVA at matched false-alarm rate) declares an anomaly window. Stage 2 extracts nine physical-consistency features in three families (residual-coupling concentration, innovation/noise structure, cross-correlation extent) and first triages the anomaly as a physical fault or a measurement-integrity anomaly. A cause branch may further label a sensor fault or an attack, or defer the decision when the received measurements are insufficient. Stage 2b runs an innovation CUSUM and a resilient innovation-saturating estimator for the stealthy-attack regime.

Figure 2 overviews the framework. Stage 1 is a calibrated MSPM detector answering “is there an anomaly?”. Stage 2, gated behind a Stage-1 alarm, extracts physical-consistency features. Stage 2a first triages the episode as a process fault or a measurement-integrity anomaly. Stage 2c attempts the lower-level sensor fault/attack cause label with a review/unknown option. Stage 2b adds a residual-innovation defense that recovers stealthy attacks invisible to the static residual and supplies the resilient estimate analyzed in Section 4.

### 3.6. Stage 1: Calibrated MSPM Detection

The primary end-to-end protocol fixes Stage 1 before examining labels. It uses CVA with past and future orders p=f=3, retains 90% canonical energy, and sets its $T^{2}$ and SPE limits jointly on an independent healthy record to a 3% pointwise FAR. An alarm indicator becomes active after three consecutive limit violations. Each benchmark episode declares samples 1–160 to be a healthy commissioning segment; monitoring and scoring begin only afterward. A length-40, stride-20 Stage-2 window is eligible only when its endpoint is an active CVA alarm. Consequently, Stage 2 never receives an oracle onset or an undetected episode. Static PCA, DPCA, and KPCA remain matched-FAR detection comparators, but they are not selected per episode and do not feed the primary Stage-2 result  [1,2,20,21].

### 3.7. Stage 2: Physical-Consistency Discrimination

For each eligible Stage-1 window, we extract a nine-dimensional physical-consistency feature vector $\phi \in R^{9}$ in three families motivated by how faults and attacks differ physically.

*F1: Residual-coupling concentration.* From the classical per-channel SPE contribution $c_{i}={\left(\Pi^{⊥}z\right)}_{i}^{2}$ and its $\ell_{1}$-normalization $\bar{c}=c/{∥c∥}_{1}$, we take the Gini coefficient of $\bar{c}$, its top-1 and top-3 mass shares, and its entropy $-\sum_{i}{\bar{c}}_{i}\log{\bar{c}}_{i}$. A channel-confined attack may concentrate residual energy on S, whereas a coupled process fault may spread it. Because projection can also diffuse a localized injection, these features are treated as candidate evidence rather than a guaranteed separator.

*F2: Innovation and noise structure.* From a one-step predictor fit on normal data we form the per-channel innovation and take its minimum variance over channels, the fraction of channels whose innovation variance falls below a normal-referenced floor, and the mean lag-one autocorrelation. A DoS/freeze attack drives innovation variance toward zero on S; replay reinstates normal-looking values with altered temporal dependence. These diagnostics follow standard innovation-whiteness and sequential-change monitoring practice [58].

*F3: Cross-correlation spatial extent.* We take the Frobenius deviation of the windowed correlation matrix from a healthy reference, ${∥{\hat{C}}_{w}-C_{0}∥}_{F}$, and the count of channels exceeding an extreme standardized threshold. Faults are spatially broad and channel attacks narrow under the working model of Assumption 2.

Before classification, every physical feature is expressed as a change from the median of that episode’s label-free commissioning windows. The same centering is applied to the raw-statistics comparator. A 500-tree random forest (minimum leaf size 2; balanced class weights) maps ϕ to a window-level probability. Five stratified outer folds hold out complete runs; an inner run split fits a Platt map without using the outer test fold. Window probabilities are averaged so that every detected held-out run contributes exactly one out-of-fold score. The uncalibrated score is primary because calibration does not improve every proper-score diagnostic. Algorithm 1 summarizes the online procedure.
**Algorithm 1** Online detect-then-discriminate monitoring  1:**Offline:** fit CVA and (μ,D,P,ℓ) on normal data; calibrate CVA limits at 3% FAR on normal_test; fit one-step predictor and healthy reference $C_{0}$; train Stage-2 random forest with complete-run folds.  2:**for** each incoming sample $y_{k}$ **do**  3:     $z_{k}\leftarrow D^{-1}\left(y_{k}-\mu \right)$; compute $T_{k}^{2},{\mathrm{SPE}}_{k}$  4:     **if** $T_{k}^{2}>T_{\alpha }^{2}$ or ${\mathrm{SPE}}_{k}>Q_{\alpha }$ for ≥3 consecutive *k* **then**  5:         mark anomaly window; extract ϕ on sliding windows  6:         center features on commissioning median; aggregate RF probabilities  7:         run innovation CUSUM (Stage 2b); update resilient estimate  8:         **output** process fault or measurement-integrity anomaly  9:         optionally output sensor fault, attack, or review/unknown + channel-concentration evidence10:     **end if**11:**end for**

### 3.8. Stage 2c: Benign-Sensor-Fault and Identifiability Protocol

The added stress test treats a benign sensor fault as an independent causal class rather than silently merging it with the simulated attacks. On healthy wind-farm simulations we generate six declared mechanisms (Table 2): calibration drift, noise inflation, stuck-at output, intermittent sample hold, quantization, and isolated spikes. There are six independently simulated runs per mechanism (36 runs total), with separate healthy seeds. These mechanism probes test coverage only; they are synthetic and are not represented as field-confirmed instrument failures.

A stricter negative control addresses causal identifiability. For each of the 12 bias, 12 replay, and 12 denial-of-service attack runs in each domain, we create a benign causal relabel—calibration bias, stale acquisition buffer, or stuck-at failure—whose received array is an exact copy of the corresponding attack array. The two labels therefore have identical commissioning values, alarm windows, physical features, and raw features. Each pair shares one equivalence-group identifier and one outer fold. The stealthy attack family has no benign matched counterpart. Distinctive sensor-fault mechanisms are added only to the wind-farm simulation because we do not posit a TEP instrumentation model beyond the exact observational-equivalence control.

The complete-run median feature vector is the evaluation unit. A class-weighted multinomial logistic model uses the nine physical features, and a class-weighted XGBoost model uses the raw run statistics. Five shuffled group folds generate one out-of-fold probability vector per detected run; the equivalence group, rather than the causal label, determines the fold. We report three-class balanced accuracy and macro-F1, with 2000 equivalence-group bootstrap replicates. A separate physical-feature logistic branch evaluates process fault versus the pooled measurement-integrity class. Within that class, the sensor-fault/attack branch reports all cause results, the exact matched-pair negative control, and a symmetric review rule: a cause decision is released only when its larger class probability exceeds a declared confidence threshold. No threshold is selected on the test folds.

### 3.9. Stage 2b: Residual-Innovation Defense for Stealthy Attacks

A model-aware adversary can shape the injection to hold SPE below $Q_{\alpha }$ (Definition 1), evading the static residual. However, a *sustained* corrupting bias perturbs the one-step innovation $\nu_{k}=z_{k}-{\hat{z}}_{k|k-1}$ (Proposition 2). We run a two-sided cumulative-sum (CUSUM) test [58] on the whitened innovation and on per-channel innovation variance (reference value $k_{c}=0.5$ and decision interval h=8.0 in standardized units, as released in toolkit.py). It is OR-combined with the Stage-1 alarm, so that an attack invisible to SPE but sustaining an innovation shift is still routed into Stage 2. The same innovation stream drives a resilient innovation-*saturating* estimator whose error is proved uniformly ultimately bounded, independent of attack magnitude, in Section 4.6. Together, Stage 2b is the control-theoretic bridge between detection and discrimination in the hardest, stealthy regime.

### 3.10. Use of Generative Artificial Intelligence

During manuscript preparation, the authors used OpenAI Codex (GPT-5 family) for English-language editing, analysis-code review, and visual-layout assistance. The tool was not used to generate study data, select the study design, or make the final scientific interpretations. The authors independently reviewed the edits, reran the released code, verified the reported results and figures, and take full responsibility for the publication.

## 4. Detectability, Causal Identifiability, and Resilient Estimation

This section answers four questions about a fault–anomaly framework: (i) How much can a stealthy attacker inject while staying under the detector? (ii) When is an attack provably undetectable by a residual monitor? (iii) When can two declared causes be identified from received measurements at all? (iv) Why can physical-consistency features separate some mechanisms, and can the resilient estimator be given a bounded-error guarantee. We label each result by the strength of its claim: *[proved]* holds under the stated linear/PCA abstraction; *[mechanistic]* is an explanation our experiments support but do not close in closed form. Boundaries of the abstraction are stated explicitly.

Throughout, recall the residual map restricted to the attack support,

(4) $$R_{S}\;=\;\Pi^{⊥}E_{S}\;\in \;R^{n\times s},$$

which is the object the whole theory turns on: it maps a standardized injection on the corrupted channels into the residual subspace that the SPE statistic monitors.

### 4.1. Covert Magnitude Budget

We state the covert budget as a definition: once the covertness constraint is written down, the bound below follows in one line from the definition of the smallest singular value. We reserve *Proposition* numbering for results whose proof requires more than unpacking a definition.

**Definition** **1** 
(Covert magnitude budget)**.** *Let an attack with support S inject the standardized corruption $u=D^{-1}E_{S}a$, and write $\tilde{a}=D_{S}^{-1}a$ for the channel-scaled injection, so that $\Pi^{⊥}u=R_{S}\;\tilde{a}$. The covert magnitude budget of support S is the largest $∥\tilde{a}∥$ compatible with keeping the residual contribution within the SPE control limit, $∥\Pi^{⊥}u∥\leq \sqrt{Q_{\alpha }}$.*

**Remark** **1** 
(Closed form for the budget)**.** *Whenever $\sigma_{\min}\left(R_{S}\right)>0$, the covert magnitude budget of Definition 1 satisfies*

(5) $$∥\tilde{a}∥\;\leq \;\frac{\sqrt{Q_{\alpha }}}{\sigma_{\min}\left(R_{S}\right)}.$$

*This is immediate: by the definition of the smallest singular value, $∥R_{S}\;\tilde{a}∥\geq \sigma_{\min}\left(R_{S}\right)\;∥\tilde{a}∥$. Combining with the covertness constraint $∥R_{S}\;\tilde{a}∥=∥\Pi^{⊥}u∥\leq \sqrt{Q_{\alpha }}$ and dividing by $\sigma_{\min}\left(R_{S}\right)>0$ gives *(5)*.*

*Interpretation.* The covert injection magnitude an attacker may apply without tripping the SPE limit is inversely proportional to $\sigma_{\min}\left(R_{S}\right)$. A larger or better-aligned support usually lowers $\sigma_{\min}\left(R_{S}\right)$ (more residual directions in which to hide), enlarging the budget. The detectability margin of the monitor against a support-S attack is thus precisely $\sigma_{\min}\left(R_{S}\right)$.

*Terminology.* We use “covert” throughout in the operational sense of an injection that stays below the deployed monitoring limit. In the resource taxonomy of [13] this is the sensor-only *stealthy* class. The *covert attack* proper of that taxonomy requires actuator resources and closed-loop cancellation, which Assumption 1 places outside our threat model.

### 4.2. The Undetectable Phase Transition (SPE Monitor)

**Proposition** **1**  (Undetectable phase transition for the SPE statistic (*proved, SPE-only*))**.** *This result concerns the SPE (squared prediction error, Q-statistic) residual monitor in isolation. Remark 2 below states precisely what happens once SPE is combined with $T^{2}$, which is the monitor actually deployed in Section 5. Let d=n−ℓ be the SPE residual-subspace dimension and s=|S|.*

1. *If s≤d, then generically (for supports S not aligned with the principal subspace) $R_{S}=\Pi^{⊥}E_{S}$ has full column rank, so $\sigma_{\min}\left(R_{S}\right)>0$; by Remark 1 the attack pays a finite, strictly positive SPE-detectability cost, and a sufficiently large injection necessarily forces the SPE over its limit. This direction is the positive guarantee of the proposition (below $s^{★}$, every support-S attack is SPE-detectable at large enough magnitude). However, we flag its quantitative content explicitly: “strictly positive” is an algebraic statement, and near the transition the margin can be so small that the covert budget *(5) *is enormous in practice. On TEP, $\sigma_{\min}\left(R_{S}\right)$ at s=d is $∼1.1\times 10^{-5}$, so the SPE monitor is already effectively blind one step* before *the transition (a covert budget of order $10^{5}$ standard deviations). On the wind farm the margin at s=d is 0.048, a meaningful but modest cost. The engineering boundary is therefore where $\sigma_{\min}\left(R_{S}\right)$ becomes small relative to plausible injection magnitudes (read off the $\sigma_{\min}$-versus-s curve of Section 5), not the algebraic collapse point itself.*
2. *If s>d, then $\mathrm{rank}(R_{S})\leq d<s$, so $R_{S}$ has a nontrivial null space: there exists $\tilde{a}\neq 0$ with $R_{S}\;\tilde{a}=0$. The corresponding injection produces* zero SPE residual contribution *at* arbitrary *magnitude: an* SPE-monitor*-undetectable attack exists. As Remark 2 shows, this same injection is generally* not *silent under the deployed $T^{2}\;\vee \;\mathrm{SPE}$ monitor, because it necessarily has a nonzero component in range(P) (the SPE-null space and the $T^{2}$-monitored subspace are complementary), so case 2 is a statement about the SPE channel specifically, not about detectability of the deployed system.*

*The SPE-only transition occurs at $s^{★}=d+1$*.

**Proof.**  Rank counting on $R_{S}$; Supplementary Materials, Section S1.    □

**Remark** **2**  (The deployed $T^{2}\;\vee \;\mathrm{SPE}$ monitor has no such transition (*proved*))**.** *The $s^{★}=d+1$ collapse above is specific to the SPE channel. Section 5 deploys Stage 1 as the OR-combination $T^{2}\;\vee \;\mathrm{SPE}$, whose combined normalized quadratic form $B_{S}=E_{S}^{⊤}ME_{S}$, with $M=P\mathrm{diag}\left(1/\lambda \right)P^{⊤}/$$t_{\lim}+\Pi^{⊥}/Q_{\alpha }$, is positive definite for* every *nonempty support of every size (a complementary-kernel argument on M; Supplementary Materials, Section S1). Hence $\sigma_{\min}$ of the combined map never vanishes and no support size admits an arbitrary-magnitude attack undetectable by the deployed monitor: an SPE-null-space injection ($\tilde{a}\in \ker\left(R_{S}\right)$) hides only the SPE component and leaves the $T^{2}$ arm quadratically amplified. The correct reading of Definition 1 and Proposition 1 is therefore not “the deployed system loses detectability past $s^{★}$,” but the narrower statement: the combined monitor is defeated only by an attacker who simultaneously evades* both *arms, and we claim no support size at which that joint evasion is guaranteed. The $s^{★}$ transition is thus a structural weakness of a* single-statistic *linear monitor—motivating the OR-combination and the innovation-based Stage 2b defense—not a vulnerability of the system as deployed.*

*Boundary.* The existence of exactly residual-silent injections echoes the classical undetectable-attack and zero-dynamics results for dynamical systems [11,12,13]. The specific content of Proposition 1 is the exact support-size threshold $s^{★}=d+1$ for the static PCA–SPE monitor. Proposition 1 is a structural weakness of the *SPE-only, linear subspace* statistic, not a turnkey real-plant attack recipe against the deployed monitor: mounting the SPE-null-space injection requires the attacker to know *P* and to corrupt more than *d* channels simultaneously. As Remark 2 shows, it does not by itself defeat the OR-combined $T^{2}\;\vee \;\mathrm{SPE}$ statistic actually used in Section 5. Nonlinear or dynamic detectors (KPCA and CVA) further reshape the effective residual geometry. We verify the SPE-only transition numerically in Section 5 on the TEP model, where the 90%-variance PCA gives *ℓ* retained components and hence a predicted transition at $s^{★}=n-\ell +1$. The realized *ℓ* and *d* and the $\sigma_{\min}\left(R_{S}\right)$-versus-*s* curve are reported there. Any figure or table that reports this curve should be read, and is henceforth captioned, as an SPE-only quantity.

### 4.3. Stealthy Direction and Its Innovation Signature

**Proposition** **2**  (Stealthy direction and innovation signature (*proved*))**.** *Among all unit-magnitude injections on support S, the one minimizing the induced SPE aligns $\tilde{a}$ with the right-singular vector of $R_{S}$ associated with $\sigma_{\min}\left(R_{S}\right)$ [proved]. For a linear one-step predictor ${\hat{z}}_{k|k-1}$ with innovation $\nu_{k}=z_{k}-{\hat{z}}_{k|k-1}$, a sustained constant injection u shifts the stationary innovation mean to $E\left[\nu_{\infty }\right]=\left(I-\Phi \right)u$ with $\Phi ={\left(I-A\left(I-K\right)\right)}^{-1}AK$, and for any Schur-stable A (with the one-step predictor A(I−K) Schur, as assumed throughout; Supplementary Materials, Section S1) this quantity is zero iff u=0: no nonzero sustained offset is innovation-silent, unconditionally [proved]. Consequently, whenever s>d and the SPE-silent set $\ker(R_{S})$ is nontrivial (Proposition 1, case 2), the set of injections that are simultaneously SPE-silent and innovation-silent is strictly smaller than the SPE-silent set: for a constant injection the two sets intersect only at u=0, so every nonzero SPE-silent sustained offset is innovation-detected. (For s≤d the generic SPE-silent set is already trivial, and the statement is vacuous.)*

**Proof.**  The first claim is the variational characterization of $\sigma_{\min}$; the second follows from the mean recursion of the filtered estimate, a push-through identity, and ker(I−Φ)=ker(I−A)={0} for Schur-stable *A*; Supplementary Materials, Section S1, including a numerical check of the closed form.    □

This motivates the innovation CUSUM of Section 3.9 recovers stealthy attacks that the static residual misses: it detects the “residual of the residual.” The result above is about a *constant, sustained* offset only; it says nothing about transient or time-varying injections, for which the innovation-silent set is in general nontrivial. *Boundary:* For a sustained constant offset the mean-innovation signature is unconditionally nonzero, so an attacker cannot make the innovation channel silent by choice of *u* alone. What an attacker *can* still do is (i) shrink the *magnitude* of $E[\nu_{\infty }]$ by choosing *u* to make $∥(I-\Phi )u∥$ small relative to $∥u∥$ (since I−Φ need not be well-conditioned), keeping the mean shift below the CUSUM’s practical detection threshold for a finite dwell time, and (ii) use a time-varying rather than constant injection, for which this proposition does not apply. A measurement-only defense therefore still has a practical, finite-sample limit even though the idealized constant-offset case admits no exact evasion. We report the stealthy catch rate as materially improved, not solved.

### 4.4. Physical-Consistency Separation: A Modeling Assumption

We state the separation premise as a *modeling assumption* rather than a proposition. The phrase “occupy separable regions” does not specify a metric and is not a theorem. The PCA projector can also spread a localized injection across residual coordinates, so residual concentration alone need not distinguish the classes. The assumption below motivates the combined feature design; its validity is evaluated empirically.

**Assumption** **2** 
(Physical-consistency separation)**.** *We assume the fault and attack classes occupy separable regions of the physical-consistency feature space of Section 3.7, along two structural axes:*

- *Coupling (F1/F3): A physical fault perturbs the plant state, which propagates through the process Jacobian to a dense set of correlated channels; its normalized residual-contribution vector $\bar{c}$ therefore has low concentration (small Gini, large entropy) and broad correlation-matrix deviation. A support-S attack injects only on S and, absent genuine physical coupling, cannot manufacture correlated residuals on unattacked channels, so $\bar{c}$ is concentrated and the correlation deviation is narrow.*
- *Noise (F2): A DoS/freeze attack collapses innovation variance on S below the physical floor that a real fault preserves; replay reinstates normal-looking values with the wrong temporal autocorrelation.*

*Boundary.* This is a feature-design assumption, not a universal mechanism. The geometry of $\Pi^{⊥}$ can make a single-channel injection appear diffuse, while a localized physical fault can remain spatially concentrated. Accordingly, the experiments evaluate the nine features jointly and do not claim that any individual family is sufficient. A feature-aware attacker with access to multiple channels may deliberately reproduce broad residual, innovation, and correlation signatures. Conversely, a local equipment or sensor fault may remain sharply concentrated. The present experiments do not establish robustness to those boundary-blurring cases; outside a plant-validated separation envelope, the appropriate output is review rather than a forced cause label.

The three feature families adapt established contribution, innovation, and correlation diagnostics to post-alarm fault–attack attribution; they are not presented as new anomaly detectors.

### 4.5. Observational Equivalence of Sensor Faults and Attacks

**Proposition** **3**  (Measurement-only causal non-identifiability (*proved*))**.** *Let C∈{sen,atk} denote the declared cause of an alarm window and let Y contain every received measurement available to a decision rule. If *

L(Y∣C=sen)=L(Y∣C=atk),

*then every deterministic or randomized measurement-only cause rule has balanced accuracy 1/2. Under equal class priors its Bayes error is 1/2, and every real-valued measurement-only score has population AUC 1/2 when ties receive half credit. In particular, exact paired copies must receive the same score from a deterministic fitted model.*

**Proof sketch.**  The rule’s probability of predicting attack is the same under both conditional laws. Its attack recall and sensor-fault specificity therefore sum to one. Identical score distributions similarly make the two pairwise orderings equiprobable. Full proof: Supplementary Material, Section S1.    □

**Remark 3** 
(Operational consequence)**.** *The proposition concerns the information in Y, not model capacity. More trees, deeper networks, or additional deterministic features of the same telemetry cannot resolve an exact causal equivalence. Identification requires an observation whose conditional law differs across causes, such as a maintenance record, sensor self-test or quality flag, redundant independent instrument, authenticated communication event, or forensic security log. Absent such evidence, review is the defensible output.*

### 4.6. Resilient Innovation-Saturating Estimator

Model the standardized normal dynamics identified on fault-free data as $z_{k+1}=Az_{k}+w_{k}$, with *A* Schur-stable and bounded process noise $∥w_{k}∥\leq \bar{w}$. We propose the innovation-*saturating* estimator

(6) $${\hat{z}}_{k+1}=A{\hat{z}}_{k}+L\;{\mathrm{sat}}_{\rho }\left(\nu_{k}\right),\;\nu_{k}=z_{k}-{\hat{z}}_{k},$$

where ${\mathrm{sat}}_{\rho }\left(\cdot \right)$ saturates the innovation at radius ρ, capping the influence of an unbounded sensor injection on S. Let $e_{k}=z_{k}-{\hat{z}}_{k}$ denote the estimation error.

**Proposition** **4**  (Uniformly ultimately bounded error (*proved under assumptions*))**.** *Suppose that, for the attacked support S, there exist S≻0 and ϵ>0 with ${\bar{A}}^{⊤}S\bar{A}-S⪯-\epsilon I$, where $\bar{A}=A-L_{:,S^{c}}\Pi_{S^{c}}$ is the clean-channel error map (a Stein/Lyapunov condition on $\bar{A}$, not on A−L: it is feasible whenever $\bar{A}$ is Schur, reduces to the usual condition on A−L when every channel is clean (S=∅), and to Schur stability of A itself when every channel is attacked ($S^{c}=\emptyset$), which holds by assumption), and suppose the uncorrupted channels satisfy the noise bound. (The condition is stated per support: a guarantee uniform over an admissible family of supports requires it for each member, a combinatorial family in general; a block-diagonal gain design collapses this to one condition per block.) Suppose further the saturation-margin (small-gain) condition of Supplementary Materials Section S1 holds, so that clean-channel innovations remain unsaturated on the invariant set. It is vacuous when $S^{c}=\emptyset$, and without it a coarser unconditional bound (Supplementary Materials, Section S1) still holds since A is Schur and ${\mathrm{sat}}_{\rho }$ is globally bounded. Then the error of (6) is uniformly ultimately bounded, with the attack-driven contribution entering only through the columns of the observer gain indexed by the attacked support,*

(7) $$\underset{k\to \infty }{lim sup}∥e_{k}∥\;\leq \;\sqrt{\frac{\lambda_{\max}\left(S\right)}{\lambda_{\min}\left(S\right)}}\;\frac{\bar{w}+∥L_{:,S}∥\;\rho \sqrt{s}}{1-\gamma },$$

*where s=|S|, γ∈(0,1) is the contraction rate the Stein condition induces on the clean-channel error dynamics $\bar{A}$ ($\gamma^{2}=1-\epsilon /\lambda_{\max}\left(S\right)$), the conditioning factor $\sqrt{\lambda_{\max}\left(S\right)/\lambda_{\min}\left(S\right)}$ equals 1 whenever S can be taken scalar (in particular in the single-channel validation of Section 5, whose published numbers are unchanged), and $L_{:,S}$ is the block of L mapping attacked-channel innovations into the full state. The bound is* independent of the attack magnitude *on S: the saturation caps each corrupted innovation coordinate at ρ, so the corrupted correction is bounded by $∥L_{:,S}∥\rho \sqrt{s}$ however large the injection. This term is a* cross-channel *corruption: the leakage onto the estimates of the* unattacked *channels $S^{c}$ is governed by the off-diagonal gain block $L_{S^{c},S}$, and vanishes iff L is block-diagonal with respect to the $S/S^{c}$ partition.*

**Proof sketch.**  Keep the attacked-channel correction whole (splitting off its clean part would make the bound circular), so the error recursion pairs $\bar{A}$ with the saturated attacked-channel term, whose norm the saturation caps at $\rho \sqrt{s}$; a Stein–Lyapunov argument on $\bar{A}$ then gives the invariant sublevel set. Full recursion and details: Supplementary Materials, Section S1). A remark there also fixes the correct instantiation of γ in the single-channel numerical validation of Section 5: with the whole (scalar) state under attack, $S^{c}=\emptyset$ forces γ=|A|, not |A−L|. With this γ the closed form strictly exceeds the empirical steady-state error at every saturation radius tested.    □

*Design tension (open problem):* The saturation radius ρ trades resilience against sensitivity: a small ρ shrinks the attack-driven error bound (7) but also clips genuine fault-induced innovations, reducing Stage-1 fault sensitivity. The worst-case-attack criterion (minimize (7)) and the empirical fault-detection criterion therefore pull ρ in opposite directions. We present the joint optimization as an open problem and, in Section 5, report the estimator’s behavior at a fixed ρ chosen to keep fault sensitivity intact.

*Cross-channel design implication:* Equation (7) isolates the attacked-support gain block $L_{:,S}$ as the sole conduit for attack-driven error, refining the single-channel view in which corruption is lumped into an undifferentiated term. Two consequences follow. First, the ultimate error scales with $∥L_{:,S}∥\sqrt{s}$, i.e., with the *number* of simultaneously attacked channels, not merely their magnitude, so a wider attack support is more damaging even under saturation, consistent with the covert-budget geometry of Definition 1. Second, the leakage onto clean-channel estimates is carried by the off-diagonal block $L_{S^{c},S}$. A gain designed to be near-block-diagonal for a suspected attack partition provably limits contamination of the unattacked estimates, at the usual cost of estimation efficiency on the clean channels. This makes precise why a whole-vector estimator that ignores which channels are under attack spreads a localized injection across the state, whereas the physical-consistency features of Section 3.5, which read the *spatial* residual pattern across channels, can remain informative because the contamination footprint is structured by $L_{:,S}$ rather than uniform.

## 5. Results

### 5.1. Evaluation Cases and Locked Protocol

The wind-farm case is a physics-based SCADA simulator with 14 channels and six equipment-fault modes: gearbox-bearing degradation, generator overheating, pitch-system fault, drivetrain imbalance, converter-cooling fault, and aerodynamic power-curve degradation. It contains 3000 healthy training samples, 3000 independent healthy calibration samples, 30 fault runs, and 48 attack runs (12 per attack family). The cross-domain case is the Tennessee Eastman Process (TEP), with 52 channels, a 500-sample healthy training record, a 960-sample independent healthy calibration record, 21 standard fault runs [2,59], and the same 48 measurement-layer attack runs.

The primary protocol is fixed before model comparison. Samples 1–160 of each episode form a declared healthy, label-free commissioning segment. A single CVA(p=3,f=3, 90% energy) detector is calibrated on the independent healthy record to a 3% pointwise FAR and triggers after three consecutive violations. Only post-commissioning windows whose endpoint carries an active alarm enter Stage 2. Features are changes from the episode’s commissioning median. Five stratified outer folds hold out complete runs, an inner complete-run split fits the optional Platt map, and each detected run contributes one out-of-fold mean probability. The primary estimate uses the raw random-forest probability. A 2000-replicate stratified run bootstrap forms AUC intervals.

### 5.2. Strict End-to-End Performance

**Figure 3:**
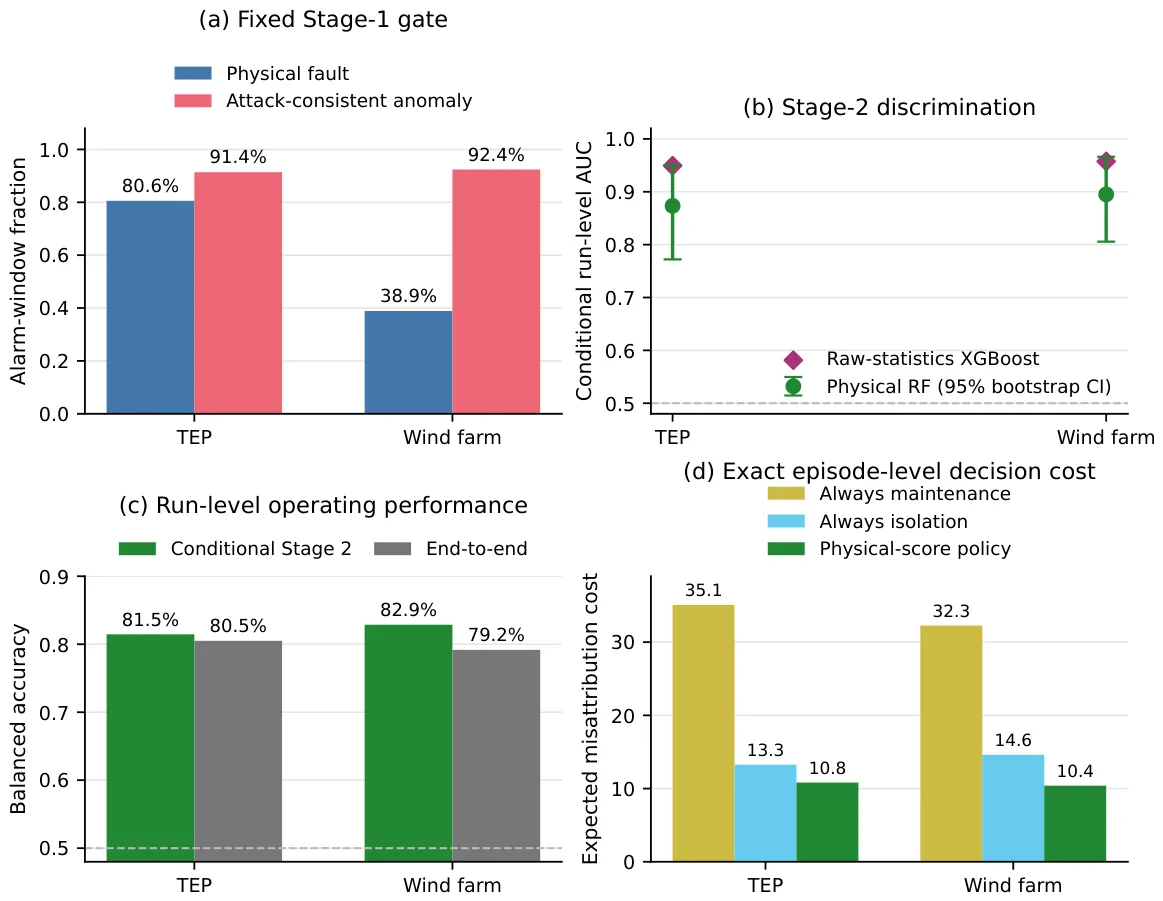
Strict run-disjoint evaluation. (**a**) Fraction of post-commissioning windows that satisfy the fixed CVA gate. (**b**) Conditional run-level AUC of the physical-feature random forest, with 95% stratified run-bootstrap intervals; raw-statistics XGBoost is shown as a clean-data comparator. (**c**) Conditional and end-to-end balanced accuracy. (**d**) Exact episode-level expected misattribution cost (EMC), including Stage-1 misses. All displayed model scores are outer-fold predictions, with one score per detected run. The dashed horizontal lines in (**b**,**c**) mark the chance level of 0.5.

Table 3 and Figure 3 distinguish three quantities that answer different questions. *Episode coverage* asks whether Stage 1 ever exposes an eligible window. *Conditional Stage-2 performance* evaluates only detected runs. *End-to-end balanced accuracy* additionally counts every missed run as an error.

TEP Stage 1 covers all 21 fault runs and 47 of 48 attack runs; the wind-farm front end covers 27 of 30 fault runs and all 48 attack runs. Coverage alone conceals prolonged blind regions. The average fraction of post-commissioning windows carrying an alarm is 0.806 for TEP faults but only 0.389 for wind-farm faults, compared with 0.914 and 0.924 for simulated attacks. In particular, the TEP alarm-window fractions for IDV3, IDV9, and IDV15 are 0.103, 0.026, and 0.308. The corresponding wind-farm values for pitch-system fault and aerodynamic degradation are 0.062 and 0.077. These are coverage limitations, not Stage-2 errors.

Conditional physical-feature AUC is 0.873 on TEP (95% run-bootstrap interval 0.772–0.950) and 0.895 on the wind farm (0.806–0.966). Conditional balanced accuracy is 0.815 and 0.829. Once Stage-1 misses are included, end-to-end balanced accuracy is 0.805 and 0.792. These lower, strictly gated estimates replace results from protocols that pooled windows across repeated random splits or admitted post-onset windows without verifying the fixed detector’s alarm state.

### 5.3. Construction-Artifact Diagnostics and Learned Comparator

A pre-commissioning source diagnostic asks whether the label can be inferred before the simulated event, after subtracting each episode’s commissioning median. Its outer-fold AUC is 0.594 on TEP and 0.637 on the wind farm, far below the post-alarm raw-statistics AUCs of 0.949 and 0.958. The values remain above 0.5, so residual source structure is acknowledged rather than dismissed.

The paired-background control removes a more specific confound. For each of 24 wind-farm seeds, fault and attack variants share the same contiguous healthy trajectory; test probabilities are aggregated once per held-out run. Balanced accuracy is 0.936 and AUC is 0.997, versus 0.942 and 0.999 for the block-resampled-background control. The −0.005 balanced-accuracy difference reduces concern that the principal post-alarm separation is caused only by background stitching. It does not substitute for field attacks.

Raw-statistics XGBoost remains the stronger clean-data ranking model: conditional AUC is 0.949 on TEP and 0.958 on the wind farm. The comparison therefore establishes a trade-off, not physical-feature superiority. The nine-feature model is lower-dimensional and directly traceable to residual concentration, innovation structure, and cross-channel coupling; the raw model is the appropriate accuracy benchmark.

### 5.4. SPE Detectability and Resilient-Estimation Checks

**Figure 4:**
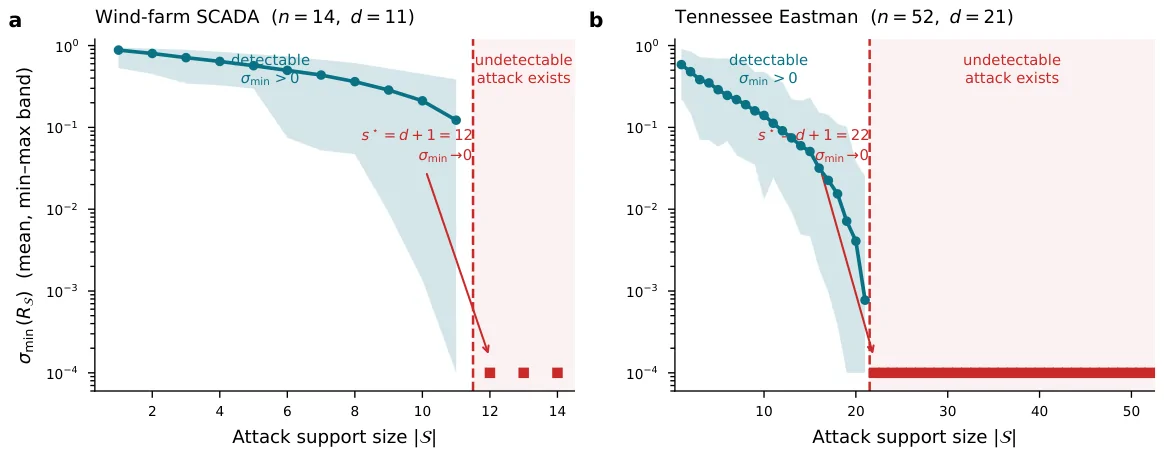
SPE-channel margin versus attack-support size. Mean and range are computed over random supports. The dashed vertical line marks the SPE-only transition $s^{★}=d+1$, beyond which an SPE-silent attack exists; the shaded region to its right is the undetectable region for the SPE channel. The algebraic boundary applies only to the SPE channel, not to the combined detector or the Stage-2 classifier.

Figure 4 evaluates Proposition 1. A 90%-variance PCA retains 31 components on TEP (n=52,d=21) and three on the wind farm (n=14,d=11). Across random supports, the SPE margin remains positive for all sampled supports with |S|≤d and becomes numerically zero at |S|=d+1. The thresholds are therefore 22 channels for TEP and 12 for the wind farm. At s=d, however, the margin is only $1.1\times 10^{-5}$ on TEP and 0.048 on the wind farm. The result characterizes the SPE channel; it is not an attribution guarantee and does not create an exact transition for the deployed $T^{2}\vee \mathrm{SPE}$ rule.

For the standardized TEP XMEAS(1) record, the fitted scalar dynamics are A=0.610 and ${\hat{\sigma }}_{w}=0.793$. With truncated simulation noise $\bar{w}=4{\hat{\sigma }}_{w}=3.172$, saturation radius ρ=2, and the fully specified protocol in Supplementary Table S1, the steady running-mean error is 3.083, below the analytical bound 11.221. The complete ρ sweep, including the declared fault step and CUSUM constants, is generated by analysis/06_theory_experiments.py.

### 5.5. Operating-Envelope Limitation

**Figure 5:**
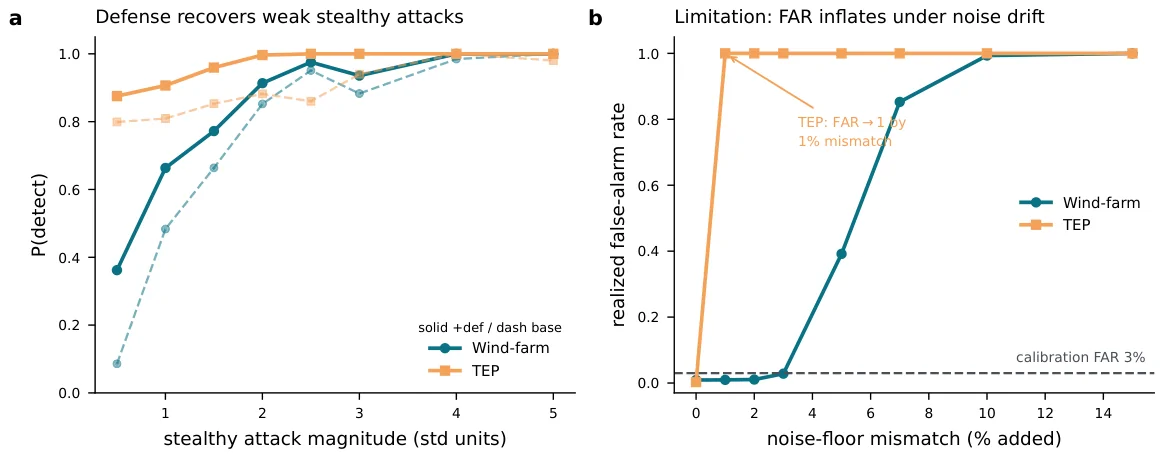
Operating-envelope diagnostics. The left panel (**a**) varies simulated attack magnitude; solid curves include the Stage-2b innovation defense and dashed curves show the static Stage-1 detector alone. The right panel (**b**) shows realized Stage-1 FAR under measurement-noise drift; the dashed horizontal line marks the calibration FAR of 3%. The figure diagnoses detector sensitivity and is not part of the strict Stage-2 model comparison.

The released stress test confirms that Stage-1 FAR can increase when the measurement-noise floor changes (Figure 5). This is a detector stationarity result. Because the earlier robustness comparison used a different resampling protocol, it is not used to rank the two Stage-2 models in the primary claims. Prospective deployment requires periodic or adaptive limit calibration and independent shift validation.

### 5.6. Field-Data Transfer and Cross-Domain Evidence

**Figure 6:**
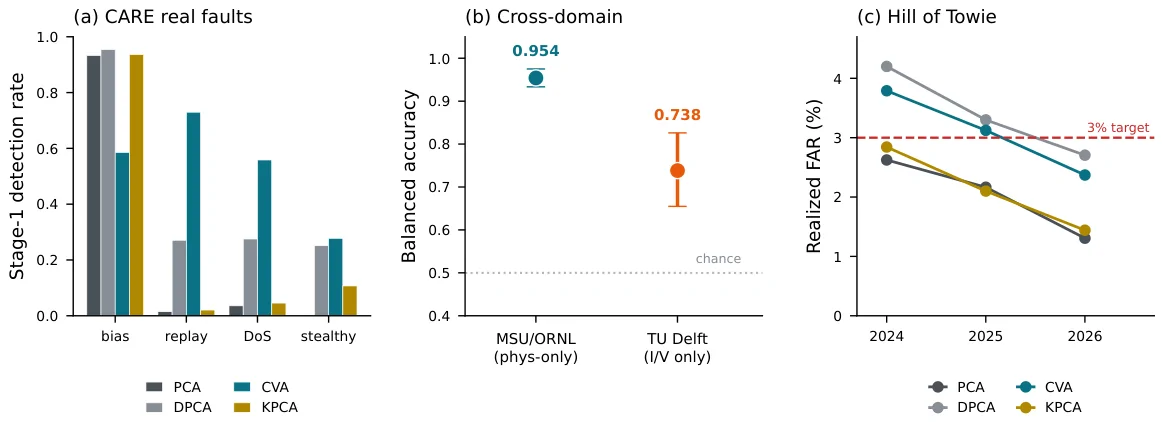
Field-data evidence. (**a**) Stage-1 detection of synthetic attack families on CARE normal telemetry at matched FAR; replay and DoS favor CVA, whereas the stealthy family is hardest. (**b**) Cross-domain discrimination with real attack labels on MSU/ORNL and TU Delft. (**c**) Annual realized FAR on Hill of Towie SCADA under limits calibrated on the first year.

No public wind-farm dataset currently combines labeled equipment faults with labeled measurement-layer cyber-attacks. The field evaluation therefore separates three evidence types: real wind-farm faults with threat-model-matched synthetic attacks, wind-specific attack testbeds, and adjacent energy-CPS datasets with real attack labels. The distinction is retained throughout; none of the results below validates attribution against real attacks on an operating wind farm. Figure 6 and Table 4 summarize the evidence type and result of each field-data evaluation.

#### 5.6.1. CARE Wind-Turbine SCADA

We use the CARE benchmark [60] across Farms A, B, and C on their common ten-channel schema. Event-log anomaly identifiers define 45 fault-leading segments from 30 turbines (12, 6, and 27 segments by farm). The prediction periods include healthy lead-in telemetry, and 11 turbines contribute more than one segment, so segments are not independent. Entire turbines are held out with GroupShuffleSplit; synthetic attacks are placed on contiguous, unshuffled real-normal backgrounds. Detector calibration and feature scaling use training-side normal files.

At per-sample FARs of 2.3–3.0%, the union of four Stage-1 monitors alarms in 43 of 45 fault segments. This is a coverage check, not a fault-versus-healthy accuracy result: the same “any run of at least three alarms” event rule also fires on 73.8% of length-matched healthy segments because the records are long. With a stricter ten-consecutive-alarm rule, the rates are 82.2% for fault segments and 50.2% for healthy segments. Subsequent attribution results are evaluated at grouped episode level and do not use this event rule as a fault classifier.

The grouped Stage-2 model retains ranking information (AUC 0.941; ten-split diagnostic interval [0.913, 0.966]) but fails at the synthetic-fit threshold τ=0.5: balanced accuracy is 0.500 because essentially every held-out episode is labeled as an attack. This result separates ranking transfer from operating-point transfer. A threshold selected from training-side labeled groups raises balanced accuracy to 0.731 but is unstable across splits. A label-free threshold set to the 0.95 quantile of scores on the plant’s training-side healthy pseudo-runs yields balanced accuracy 0.813, fault recall 0.969, and attack recall 0.658. Quantile sensitivity is monotone: q=0.90 gives balanced accuracy 0.858 and attack recall 0.777, whereas q=0.99 gives 0.666 and 0.338. The chosen q=0.95 is therefore an illustrative plant anchor, not a universally optimal value.

Attack recall remains conditional on the training library. When one synthetic family is excluded from training, held-out DoS recall falls from 0.94 to 0.10 and bias recall from 0.84 to 0.60; replay and stealthy recall are low even when represented. The CARE experiment supports plant-normal anchoring and demonstrates score transfer, but it does not establish robustness to unseen attack mechanisms or real wind-farm attacks.

#### 5.6.2. Auxiliary and Cross-Domain Datasets

Three wind datasets test narrower properties. On EDP SCADA [61], the dominant residual channels associated with the synthetic families map to physically meaningful named signals. This is an interpretability audit, not an evaluation of top-*k* channel accuracy or exact support recovery. Hill of Towie SCADA [62] keeps annual realized FAR between 1.3% and 4.2% under limits calibrated on the first year. On the ELECTRON/KIOS SUC1 testbed [63], Stage 1 detects three of four wind-specific attack events; these records do not pair real attacks with real equipment faults.

The MSU/ORNL power-system dataset [14] provides real attacks and real natural events. Excluding no-event segments gives 30 attack and 30 physical-event runs; grouped measurement-only discrimination reaches balanced accuracy 0.954 (95% interval [0.933, 0.975]) and AUC 0.995. The TU Delft IEC-61850 dataset [64] is harder: balanced accuracy is 0.738 (AUC 0.871), unchanged when restricted to current and voltage channels apart from an AUC reduction to 0.850. These cross-domain results support the general feasibility of measurement-only event–attack discrimination, not transfer of the wind-specific feature model.

### 5.7. Interpretation of the Learned Baseline

The strict comparison holds the alarm gate, commissioning centering, outer run folds, and run-level aggregation fixed while changing the Stage-2 representation and classifier. Raw-statistics XGBoost achieves conditional AUC 0.949 on TEP and 0.958 on the wind farm, compared with 0.873 and 0.895 for the physical-feature random forest. Thus, the evidence does not support a claim that the compact representation is the best clean-data classifier.

Because representation and learning algorithm change together, this is a comparison between two complete pipelines; it does not isolate a causal representation effect or a classifier effect. The locked evidence also does not include the requested representation-by-classifier factorial benchmark or F1/F2/F3 family ablations. We therefore make no claim about which feature family contributes most for a given anomaly mechanism or which feature can be removed without material loss.

Secondary noise and drift grids document model sensitivity under a different repeated-split protocol and are not mixed with the strict headline estimates. A robustness comparison between representations requires a prespecified shift family, complete-run outer folds, and fresh evaluation data. The present benefit of the physical representation is auditability: every input corresponds to a declared residual, innovation, or coupling diagnostic.

### 5.8. Cross-Fitted Probability Calibration

Within each outer fold, a complete-run inner split fits a logistic (Platt) map to run-aggregated probabilities; the outer test runs are never used to fit the classifier or calibrator. Table 5 reports all proper-score diagnostics rather than selecting the favorable ones.

Calibration changes the diagnostics in mixed directions. It slightly improves TEP Brier score and both log losses, but worsens TEP ECE, wind-farm Brier score, and balanced accuracy on both datasets. The raw out-of-fold score is therefore retained for the primary operating result. The calibrated scores are released for inspection but do not support a general probability-calibration claim or a validated reject policy.

Plant-specific recalibration is therefore an operating-point adjustment, not a robustness guarantee. It is appropriate only when the deployed score retains useful ordering and the data-quality conditions fall within a validated envelope. Missingness, asynchronous sampling, topology or control-mode changes, and noise that changes the feature ordering require revalidation or model revision. The present study does not report missing-data or irregular-sampling stress tests, so those conditions remain outside the validated evidence.

### 5.9. Benign-Sensor-Fault and Causal-Identifiability Stress Test

The extended protocol adds a declared benign sensor-fault class without changing the locked primary binary estimates. Stage 1 detected all 36 matched sensor-fault runs on TEP and all 72 sensor-fault runs on the wind-farm simulation (36 exact matched copies plus 36 distinctive mechanisms). It also detected 21/21 and 27/30 process-fault runs and 47/48 and 48/48 attack runs, respectively. All subsequent metrics use one alarm-gated, out-of-fold vector per detected run.

Table 6 separates the questions that the stress test can and cannot answer. The physical-feature hierarchical branch distinguishes a process fault from the pooled measurement-integrity class with AUC 0.820 on TEP and 0.853 on the wind-farm simulation. The harder three-class task remains modest: physical-feature balanced accuracy is 0.543 (95% equivalence-group bootstrap interval 0.459–0.622) on TEP and 0.535 (0.458–0.606) on the wind-farm simulation. Raw XGBoost reaches 0.522 and 0.593, respectively. These estimates do not support autonomous three-way cause attribution.

**Figure 7:**
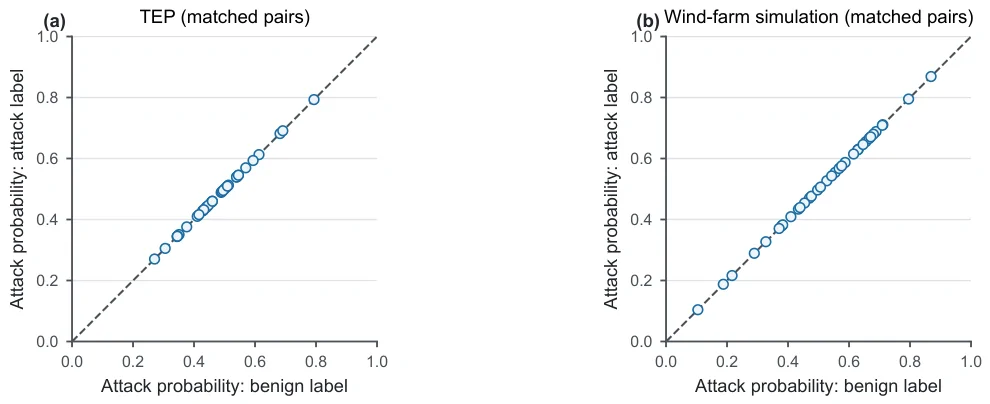
Exact observational-equivalence control for the physical-feature cause branch. Each marker compares the out-of-fold attack probability assigned to the benign sensor-fault label (horizontal axis) and to the attack label (vertical axis) for one identical received trajectory. Bias/calibration bias, replay/stale buffering, and denial-of-service freezing/stuck-at failure share both their measurements and their equivalence-group fold. Equality is therefore required; neither axis is a field-derived causal probability.

The exact causal-relabel pairs give the decisive boundary result. Figure 7 plots the out-of-fold attack probability of each benign-labeled trajectory against that of its attack-labeled copy. Every point lies on the identity line to machine precision (maximum difference $2.22\times 10^{-16}$ on TEP and zero on the wind-farm simulation for the physical model). Matched cause AUC and balanced accuracy are both 0.500 for the physical model and XGBoost. This is the required negative-control outcome under Proposition 3, not an optimization failure. A model reporting high matched-pair cause accuracy would reveal label or fold leakage.

This exact-equivalence control establishes only the chance-level endpoint. It does not determine how AUC changes as two received trajectories become nearly, rather than exactly, equivalent. A leakage-safe perturbation sweep with all variants of an underlying run kept in one outer fold would be required to estimate an AUC-versus-similarity curve. One prespecified scale-free trajectory distance for such a study is $d_{\alpha }={∥Y^{\left(\alpha \right)}-Y^{\left(0\right)}∥}_{F}/\left({∥Y^{\left(0\right)}∥}_{F}+\epsilon \right)$, where $Y^{\left(0\right)}$ is the exact matched trajectory and α controls a physically defined perturbation. Because the artifacts needed for that new experiment are not present in the current revision package, we do not report or infer an unverified transition curve.

The 36 distinctive wind-farm sensor-fault runs show why unmatched mechanism accuracy cannot establish causal identifiability: physical features recover 77.8% and raw XGBoost 88.9% of these benign runs, yet both models remain at chance on exact matched causes. At a prespecified cause-confidence threshold of 0.75, the physical model reviews 86.1% of wind matched pairs and releases decisions for 11.7% of all wind measurement-integrity runs, with 64.3% accuracy among released decisions. The low coverage is disclosed rather than converted into an inflated headline score. The complete confusion matrices and threshold sweep are reported in Supplementary Materials, Section S6.

## 6. Risk-Aware Decision Consequences

### 6.1. Consequence Model

For a detected episode with true class y∈{fault,anomaly}, the operator chooses d∈{maintenance,isolation}. The illustrative consequence matrix is

(8) $$C=\left(\begin{array}{cc} 1 & 30\\ 50 & 5 \end{array}\right),$$

where rows are true classes and columns are responses. Thus, isolating a physical fault is penalized 30, and routing an attack-consistent anomaly to maintenance is penalized 50. These are normalized severity weights, not monetary estimates or values elicited from an operating wind farm.

If *p* were a calibrated anomaly probability, posterior expected cost gives

(9) $$\tau_{\mathrm{Bayes}}=\frac{30-1}{\left(30-1)+(50-5\right)}=0.392.$$

We apply this threshold to the raw outer-fold score as a transparent cost-sensitive rule; because Section 5.8 does not establish calibration, the resulting EMC is an empirical policy comparison, not a Bayes-optimality claim.

### 6.2. Exact Episode-Level Accounting

The evaluation retains all 69 TEP and 78 wind-farm episodes (Table 7). A detected run incurs the matrix entry for the selected response. A Stage-1 miss cannot be routed correctly and is conservatively charged the corresponding off-diagonal cost. The two blanket comparators likewise operate only after an alarm, so they receive the same miss charge. No class-average constants are substituted for individual episodes.

The physical-score policy reduces EMC by 18.4% on TEP and 28.9% on the wind farm relative to the lower-cost blanket response. Nested Platt calibration would change these values to 8.64 and 11.14, respectively; the opposing movement reinforces the decision to keep uncalibrated scores primary. The comparison demonstrates how an attribution score can affect routing under one declared consequence matrix. A deployment claim requires site-specific class prevalence, response interference, and consequences to be elicited and tested prospectively.

## 7. Discussion

### 7.1. What the Strict Evaluation Establishes

The fixed-gate experiment shows that compact physical-consistency features retain useful post-alarm information under complete-run separation. Conditional AUC is 0.873 on TEP and 0.895 on the wind farm, while end-to-end balanced accuracy is 0.805 and 0.792 after Stage-1 misses are counted. These estimates are lower than the earlier repeated-split values and are more relevant to deployment because an episode cannot reach Stage 2 unless the locked CVA gate actually alarms.

Three diagnostics qualify the result. First, raw-statistics XGBoost ranks the clean simulated episodes more accurately (AUC 0.949 and 0.958); physical features provide a shorter evidence chain, not a universal accuracy gain. Second, pre-commissioning source AUC remains modestly above chance (0.594 and 0.637). Commissioning centering removes most construction information but not all of it. Third, the paired-background wind-farm control retains high run-level performance with only a 0.005 balanced-accuracy decrease, reducing concern about one specific stitching artifact. None of these controls proves field validity.

The benign-sensor-fault extension sharpens that boundary. The hierarchical physical-feature branch retains useful process-fault versus measurement-integrity ranking (AUC 0.820 on TEP and 0.853 on the wind-farm simulation), but three-class balanced accuracy is only 0.543 and 0.535. Distinctive synthetic sensor mechanisms can be recognized, whereas exact sensor-fault/attack copies necessarily receive the same score and matched cause AUC 0.500. Selective review exposes this missing information but cannot create it.

The theoretical results operate at different layers. Proposition 1 characterizes exact SPE-channel geometry, and Proposition 4 bounds an innovation-saturating estimator under declared assumptions. Proposition 3 proves why exact sensor-fault/attack causes are not identifiable from received measurements. The empirical security output is consequently described as attack-consistent rather than cyber-confirmed.

### 7.2. Field Transfer and Decision Meaning

CARE defines a stricter deployment boundary. The grouped analysis retains ranking information (AUC 0.941) but fails at the synthetic threshold. Plant-normal anchoring gives balanced accuracy 0.813, fault recall 0.969, and attack recall 0.658. Recall also falls sharply for some held-out synthetic families. This supports label-free plant anchoring as a candidate calibration step, but not out-of-the-box attribution or proof against real wind-farm attacks.

At the illustrative consequence matrix, exact episode accounting gives EMC 10.83 on TEP and 10.40 on the wind farm, reductions of 18.4% and 28.9% from the better blanket response. These values include Stage-1 misses. Calibration changes cost in opposite directions across the two domains, so the raw score remains primary. Site-level use would require operators to elicit consequences, class prevalence, and whether maintenance and isolation can be dispatched simultaneously.

### 7.3. Generalizability and Deployment Boundary

Generalizability should be interpreted at the level of structure rather than fixed operating points. The detection-gated separation of detection, attribution, and response is portable in principle, as is the non-identifiability result for observationally equivalent causes. In contrast, feature distributions, thresholds, calibration maps, and cost-optimal actions are plant- and sensor-layout-specific. The CARE threshold-transfer result and leave-one-attack-family stress tests show that ranking transfer does not imply operating-point transfer or coverage of unseen mechanisms. The current study does not include a leave-one-benign-sensor-fault analysis, so an unseen benign mechanism must not be assigned a confirmed cause; review/unknown is the safe disposition. The experiments support cross-domain feasibility, not universal thresholds, prospective field effectiveness, or complete mechanism coverage.

### 7.4. Limitations and Priorities

Ten limitations delimit the contribution. *First*, no public dataset used here pairs real wind-farm equipment faults with real measurement-layer cyber-attacks. CARE combines real faults with synthetic attacks. *Second*, the wind-farm simulator simplifies wakes, controller behavior, and fault progression. *Third*, the added benign sensor faults are synthetic. The exact matched pairs establish non-identifiability and the six distinctive mechanisms test coverage, but neither provides field sensor-fault prevalence or validity. *Fourth*, weak Stage-1 modes and noise-dependent FAR constrain pipeline coverage. *Fifth*, the small number of independent runs does not support a strong probability-calibration or reject-option claim. *Sixth*, the evaluated measurement anomaly is introduced on the supervisory telemetry path after the controller feedback branch; the threat model excludes controller-input corruption, actuator attacks, and coordinated closed-loop manipulation. *Seventh*, exact matched pairs establish the non-identifiable endpoint, but no near-equivalence AUC curve is available. *Eighth*, channel-concentration descriptors are not validated channel, subsystem, or support localizers. *Ninth*, the physical-feature/random-forest versus raw-statistics/XGBoost result confounds representation and classifier, and no feature-family ablation is claimed. *Tenth*, asynchronous sampling, missing data, and feature-aware distributed attacks have not been stress-tested.

Priorities are prospective validation of the three-way taxonomy on field sensor faults; integration of maintenance records, sensor-quality flags, redundant instruments, and authenticated security logs; plant-normal calibration; independent shift validation; genuine wind-farm attack telemetry; and site-specific consequence elicitation. A second line of work should jointly tune detection and innovation saturation so that defensive estimation does not suppress incipient-fault evidence.

## 8. Conclusions

This study evaluates an auditable triage layer placed behind a fixed multivariate sensor alarm. Under a declared commissioning period, alarm-window gating, five complete-run outer folds, and one out-of-fold score per detected episode, the nine-feature model achieves conditional AUC 0.873 on TEP and 0.895 on a wind-farm simulation. End-to-end balanced accuracy is 0.805 and 0.792 after missed alarms are counted. Exact episode accounting at the declared consequence matrix reduces cost by 18.4% and 28.9% relative to the better blanket response.

Several limits remain. Raw-statistics XGBoost has higher clean-data AUC, probability calibration is mixed, and pre-commissioning source diagnostics remain modestly above chance. A paired-background control reduces one construction concern but cannot replace field evidence. CARE telemetry shows that ranking can transfer while a synthetic threshold fails, and that plant-normal anchoring still leaves limited recall for unseen mechanisms.

The independent benign-sensor-fault stress test adds a necessary third class and reveals the central identifiability boundary. Process fault versus pooled measurement-integrity AUC is 0.820 on TEP and 0.853 on the wind-farm simulation, whereas exact sensor-fault/attack copies yield cause AUC 0.500 for both physical features and XGBoost. Distinctive mechanism accuracy does not override that result; observationally equivalent causes require side information or review.

The framework should therefore be used, if at all, as decision support that labels an episode as a physical fault or a measurement-integrity anomaly and attempts a lower-level cause only when corroborating evidence exists. It is not evidence of cyber causation and not an autonomous safety certificate. The reported concentration descriptors are not validated channel or support localizers, and no universal robustness claim is made for feedback-path corruption, unseen benign faults, near-equivalent causes, missing or irregular samples, or feature-aware distributed attacks. A high-confidence deployment claim requires field sensor faults, prospective calibration, independent shift testing, site-specific response costs, maintenance/security side information, and genuine wind-farm cyber-attacks.

## Figures and Tables

**Table 1:** Positioning against representative fault–attack attribution and monitoring studies.

Work	Domain	Real Fault–Attack Attribution	Measurement Only	UQ	Cost	Theory
Hink/Pan [14,45]	Power testbed	✓	×	×	×	×
Roy et al. [18]	Actuation layer	–	–	×	×	✓
Xue et al. [48]	CPS control loop	–	–	×	×	✓
Vaddi et al. [47]	Nuclear	–	×	×	✓	×
Zheng et al. [40]	Wind SCADA	×	✓	×	×	×
**This work**	**Wind SCADA + TEP**	×	✓	✓	✓	✓

Notes: “Real fault–attack attribution” requires both classes to be recorded physical events; this work uses real wind faults with synthetic wind attacks, so it is not credited in that column. “Measurement only” excludes relay logs and a separate cyber-event stream. UQ denotes episode-level confidence with deferral; cost denotes a consequence-weighted decision layer. ✓ indicates that the capability is provided and × that it is not; “–” indicates that the criterion is not applicable or not reported. The bold row denotes the present work.

**Table 2:** Operational taxonomy of the six synthetic benign sensor-fault mechanisms. Hard/soft severity is reported together with temporal pattern and mathematical form because a single binary label does not describe intermittent or impulsive faults completely.

Mechanism	Severity	Temporal Pattern	Representative Received-Signal Form
Calibration drift	Soft	Gradual or persistent	$y_{k}=x_{k}+b_{k}+v_{k}$ with slowly varying $b_{k}$ (or gain-like drift)
Noise inflation	Soft precision loss	Persistent/time-varying	$y_{k}=x_{k}+\sigma_{k}v_{k}$, $\sigma_{k}$ above its nominal level
Stuck-at output	Hard when complete	Persistent constant	$y_{k}=c$ on the affected channel
Intermittent sample hold	Hard during each hold	Intermittent	$y_{k}=y_{k-1}$ during declared hold intervals
Quantization	Soft resolution loss	Persistent nonlinear	$y_{k}=Q_{\Delta }\left(x_{k}+v_{k}\right)$
Isolated spikes	Soft/impulsive unless saturated	Intermittent impulsive	$y_{k}=x_{k}+o_{k}+v_{k}$ with sparse outlier sequence $o_{k}$

Notes: This is a study-specific operational mapping. It does not claim complete coverage of field instrumentation failures or universal agreement on hard/soft terminology.

**Table 3:** Strict run-disjoint validation. Stage 2 is evaluated only on runs with at least one fixed-CVA alarm window. Parentheses give 95% stratified run-bootstrap intervals for AUC.

Metric	TEP	Wind Farm
Stage-1 episode coverage, fault (%)	100.0	90.0
Stage-1 episode coverage, anomaly (%)	97.9	100.0
Alarm-window fraction, fault (%)	80.6	38.9
Alarm-window fraction, anomaly (%)	91.4	92.4
Conditional physical-feature AUC	0.873 (0.772–0.950)	0.895 (0.806–0.966)
Conditional physical-feature balanced accuracy	0.815	0.829
End-to-end balanced accuracy	0.805	0.792
Pre-commissioning source AUC	0.594	0.637
Exact episode-level EMC	10.83	10.40
Reduction versus best blanket action (%)	18.4	28.9

**Table 4:** Scope and result of each field-data evaluation.

Dataset	Evidence Type	Result
CARE A/B/C	Real wind faults; synthetic attacks	AUC 0.941; balanced accuracy 0.500 at τ=0.5 and 0.813 after plant-normal anchoring (fault/attack recall 0.969/0.658)
EDP; Hill of Towie	Wind interpretability and FAR stability	Named-channel interpretability audit (not localization accuracy); annual FAR 1.3–4.2%
SUC1	Wind-specific attack testbed	Stage-1 detects 3/4 attack events
MSU/ORNL	Real attacks and physical events	Balanced accuracy 0.954 [0.933, 0.975]; AUC 0.995
TU Delft	Real substation attacks	Balanced accuracy 0.738; AUC 0.871

**Table 5:** Outer-fold run-level diagnostics before and after nested Platt calibration. Lower is better for Brier score, log loss, and ECE.

Dataset	Score	Brier	Log Loss	ECE	Bal. Acc.
TEP	Raw	0.120	0.382	0.082	0.815
	Platt	0.116	0.377	0.097	0.775
Wind farm	Raw	0.120	0.386	0.109	0.829
	Platt	0.122	0.378	0.103	0.802

**Table 6:** Run-level benign-sensor-fault stress test. Confidence intervals are 2000-replicate equivalence-group bootstrap intervals. The process-versus-integrity branch uses physical features; matched cause AUC uses exact sensor-fault/attack copies with different causal labels.

Metric	TEP		Wind-Farm Simulation	
	Physical	Raw XGBoost	Physical	Raw XGBoost
Three-class balanced accuracy	0.543	0.522	0.535	0.593
95% interval	[0.459, 0.622]	[0.445, 0.593]	[0.458, 0.606]	[0.518, 0.669]
Three-class macro-F1	0.530	0.515	0.497	0.599
End-to-end macro recall	0.539	0.517	0.513	0.574
Process fault vs. measurement integrity AUC	0.820	—	0.853	—
Matched sensor fault vs. attack AUC	0.500	0.500	0.500	0.500
Distinctive sensor-fault recall	—	—	0.778	0.889

Notes: Classes are process fault, benign sensor fault, and simulated attack. “Physical” denotes class-weighted multinomial logistic regression on the nine run-median physical features; the raw comparator is class-weighted XGBoost. Distinctive mechanisms were generated only for the wind-farm simulation. Dashes denote analyses not run by the locked protocol, not missing favorable results.

**Table 7:** Exact episode-level expected misattribution cost at the illustrative consequence matrix. Lower is better.

Policy	TEP	Wind Farm
Always maintenance after alarm	35.09	32.27
Always isolation after alarm	13.26	14.62
Physical-score policy	10.83	10.40
Reduction versus better blanket response	18.4%	28.9%

## Data Availability

The public datasets analyzed in this study are available from their original repositories: CARE, EDP wind-turbine SCADA, Hill of Towie SCADA, ELECTRON/KIOS SUC1, MSU/ORNL power-system attacks, TU Delft IEC-61850, and the Tennessee Eastman Process benchmark. Dataset citations, repository links, and licenses are provided in the manuscript and the accompanying data cards. The wind-farm simulator, synthetic measurement-layer attack generator, analysis code, benign-sensor-fault generator, exact observational-equivalence pairs, strict-protocol harnesses, out-of-fold probabilities, and frozen result checkpoints are provided as Supplementary Materials with the submission. All added benign sensor faults are explicitly synthetic; no field sensor-fault or cyber-causation claim is made from them.

## Supplementary Materials

The following supporting information can be downloaded at https://www.mdpi.com/article/10.3390/s26175585/s1, Section S1: Proofs and extended remarks for the detectability theory; Section S2: Strict learned-model comparison; Section S3: Strict protocol disaggregated by mode; Section S4: Calibration, cost, and sensitivity protocols; Section S5: Auxiliary real-data evidence from EDP, Hill of Towie, and ELECTRON/KIOS SUC1; Section S6: Benign-sensor-fault and identifiability stress test; Figure S1: Out-of-fold three-class confusion matrices for the nine physical-consistency features; Figure S2: Coverage–accuracy trade-off for selective cause decisions; Table S1: Sensitivity of the scalar resilient-estimator validation to the innovation-saturation radius ρ; Table S2: Conditional outer-fold run-level AUC under the locked protocol; Table S3: Attack-family coverage and conditional recall at threshold 0.5; Table S4: Wind-farm fault-mode Stage-1 coverage, alarm-window fraction, and end-to-end recall; Table S5: Strict outer-fold calibration diagnostics; Table S6: Exact episode-level EMC, including all detector misses; Table S7: Stage-1 run detection in the sensor-fault extension; Table S8: Selective sensor-fault/attack cause branch at threshold t=0.75.

## Abbreviations

The following abbreviations are used in this manuscript:

CPS	Cyber-physical system
SCADA	Supervisory control and data acquisition
MSPM	Multivariate statistical process monitoring
PCA/DPCA/KPCA	Principal/dynamic principal/kernel principal component analysis
CVA	Canonical-variate analysis
SPE	Squared prediction error
CUSUM	Cumulative sum
FAR	False-alarm rate
EMC	Expected misattribution cost
UUB	Uniformly ultimately bounded
TEP	Tennessee Eastman Process
RF	Random forest
OOF	Out-of-fold
AUC	Area under the receiver-operating-characteristic curve

## References

[1] Ku W., Storer R.H., Georgakis C. (1995). Disturbance detection and isolation by dynamic principal component analysis. Chemom. Intell. Lab. Syst..

[2] Russell E.L., Chiang L.H., Braatz R.D. (2000). Fault detection in industrial processes using canonical variate analysis and dynamic principal component analysis. Chemom. Intell. Lab. Syst..

[3] Venkatasubramanian V., Rengaswamy R., Kavuri S.N., Yin K. (2003). A review of process fault detection and diagnosis: Part III: Process history based methods. Comput. Chem. Eng..

[4] Yin S., Ding S.X., Xie X., Luo H. (2014). A Review on Basic Data-Driven Approaches for Industrial Process Monitoring. IEEE Trans. Ind. Electron..

[5] Yuan S., Reniers G., Yang M. (2024). Integrated management of safety and security barriers in chemical plants to cope with emerging cyber-physical attack risks under uncertainties. Reliab. Eng. Syst. Saf..

[6] Wang Z., Wang J., Wei Z., Ye W., Zhang L. (2026). Safety integrity level assessment for safety instrumented system in oil and gas station with cyber threat. Reliab. Eng. Syst. Saf..

[7] Ji C., Sun W. (2022). A Review on Data-Driven Process Monitoring Methods: Characterization and Mining of Industrial Data. Processes.

[8] Badihi H., Zhang Y., Jiang B., Pillay P., Rakheja S. (2022). A Comprehensive Review on Signal-Based and Model-Based Condition Monitoring of Wind Turbines: Fault Diagnosis and Lifetime Prognosis. Proc. IEEE.

[9] Wang S., Vidal Y., Pozo F. (2026). Recent advances in wind turbine condition monitoring using SCADA data: A state-of-the-art review. Reliab. Eng. Syst. Saf..

[10] Liu Y., Ning P., Reiter M.K. (2011). False data injection attacks against state estimation in electric power grids. ACM Trans. Inf. Syst. Secur..

[11] Pasqualetti F., Dorfler F., Bullo F. (2013). Attack Detection and Identification in Cyber-Physical Systems. IEEE Trans. Autom. Control.

[12] Fawzi H., Tabuada P., Diggavi S. (2014). Secure Estimation and Control for Cyber-Physical Systems Under Adversarial Attacks. IEEE Trans. Autom. Control.

[13] Teixeira A., Shames I., Sandberg H., Johansson K.H. (2015). A secure control framework for resource-limited adversaries. Automatica.

[14] Pan S., Morris T., Adhikari U. (2015). Developing a Hybrid Intrusion Detection System Using Data Mining for Power Systems. IEEE Trans. Smart Grid.

[15] Amin B.M.R., Hossain M.J., Anwar A., Zaman S. (2021). Cyber Attacks and Faults Discrimination in Intelligent Electronic Device-Based Energy Management Systems. Electronics.

[16] Gupta K., Sahoo S., Mohanty R., Panigrahi B.K., Blaabjerg F. (2023). Distinguishing Between Cyber Attacks and Faults in Power Electronic Systems–A Noninvasive Approach. IEEE J. Emerg. Sel. Top. Power Electron..

[17] Semertzis I., Goyel H., Rajkumar V.S., Presekal A., Stefanov A., Palensky P. (2024). Towards Real-Time Distinction of Power System Faults and Cyber Attacks on Digital Substations Using Cyber-Physical Event Correlation. Proceedings of the 2024 12th Workshop on Modeling and Simulation of Cyber-Physical Energy Systems (MSCPES).

[18] Roy T., Dey S. (2024). On Distinguishability of Anomalies as Physical Faults or Actuation Cyberattacks. ASME Lett. Dyn. Syst. Control.

[19] Chen T., Guestrin C. (2016). XGBoost: A Scalable Tree Boosting System. KDD ’16: Proceedings of the 22nd ACM SIGKDD International Conference on Knowledge Discovery and Data Mining.

[20] Pilario K.E.S., Cao Y. (2018). Canonical Variate Dissimilarity Analysis for Process Incipient Fault Detection. IEEE Trans. Ind. Inform..

[21] Lee J.M., Yoo C., Choi S.W., Vanrolleghem P.A., Lee I.B. (2004). Nonlinear process monitoring using kernel principal component analysis. Chem. Eng. Sci..

[22] Narasimhan S., El-Farra N.H., Ellis M.J. (2022). Detectability-based controller design screening for processes under multiplicative cyberattacks. AIChE J..

[23] Yin S., Ding S.X., Haghani A., Hao H., Zhang P. (2012). A comparison study of basic data-driven fault diagnosis and process monitoring methods on the benchmark Tennessee Eastman process. J. Process Control.

[24] Wei Y., Chen Z., Ye Z.S., Pan E. (2024). High-dimensional process monitoring under time-varying operating conditions via covariate-regulated principal component analysis. Reliab. Eng. Syst. Saf..

[25] Mo Y., Sinopoli B. (2009). Secure control against replay attacks. Proceedings of the 2009 47th Annual Allerton Conference on Communication, Control, and Computing (Allerton).

[26] Giraldo J., Urbina D., Cardenas A., Valente J., Faisal M., Ruths J., Tippenhauer N.O., Sandberg H., Candell R. (2018). A Survey of Physics-Based Attack Detection in Cyber-Physical Systems. ACM Comput. Surv..

[27] Zhang J., Zio E., Ma C., Liu K., Wang W. (2025). A probabilistic cost-benefit analysis approach for cyberattack path evaluation. Reliab. Eng. Syst. Saf..

[28] Tang D., Fang Y.P., Zio E. (2023). Vulnerability analysis of demand-response with renewable energy integration in smart grids to cyber attacks and online detection methods. Reliab. Eng. Syst. Saf..

[29] Zheng H., Li X., Li F. (2025). Unsupervised cyberattack detection in smart grids: A novel approach integrating horizontal federated learning for the control center and substations. Reliab. Eng. Syst. Saf..

[30] Zhang B., Du M., Zheng H., Liu K., Lu B. (2026). Detection framework for bidirectional false data injection attacks to enhance reliability in cyber-physical power systems. Reliab. Eng. Syst. Saf..

[31] Wu W., Zhang L., Fu H., Wang K., Li X. (2022). Safety Impact Analysis Considering Physical Failures and Cyber-Attacks for Mechanically Pumped Loop Systems (MPLs). Sensors.

[32] Sztyber-Betley A., Syfert M., Kościelny J.M., Górecka Z. (2023). Controller Cyber-Attack Detection and Isolation. Sensors.

[33] Zou X., Liu W., Huo Z., Wang S., Chen Z., Xin C., Bai Y., Liang Z., Gong Y., Qian Y. (2023). Current Status and Prospects of Research on Sensor Fault Diagnosis of Agricultural Internet of Things. Sensors.

[34] Xing W., Shen J. (2024). Security Control of Cyber–Physical Systems under Cyber Attacks: A Survey. Sensors.

[35] Shoukry Y., Tabuada P. (2016). Event-Triggered State Observers for Sparse Sensor Noise/Attacks. IEEE Trans. Autom. Control.

[36] Forti N., Battistelli G., Chisci L., Sinopoli B. (2019). Joint attack detection and secure state estimation of cyber-physical systems. Int. J. Robust. Nonlinear Control.

[37] Ye L., Zhu F., Zhang J. (2020). Sensor attack detection and isolation based on sliding mode observer for cyber-physical systems. Int. J. Adapt. Control Signal Process..

[38] Schlechtingen M., Santos I.F., Achiche S. (2013). Wind turbine condition monitoring based on SCADA data using normal behavior models. Part 1: System description. Appl. Soft Comput..

[39] Bindingsbø O.T., Singh M., Øvsthus K., Keprate A. (2023). Fault detection of a wind turbine generator bearing using interpretable machine learning. Front. Energy Res..

[40] Zheng M., Man J., Wang D., Chen Y., Li Q., Liu Y. (2023). Semi-supervised multivariate time series anomaly detection for wind turbines using generator SCADA data. Reliab. Eng. Syst. Saf..

[41] Staggs J., Ferlemann D., Shenoi S. (2017). Wind farm security: Attack surface, targets, scenarios and mitigation. Int. J. Crit. Infrastruct. Prot..

[42] Viasat Inc. (2022). KA-SAT Network Cyber Attack Overview. Viasat Corporate Incident Statement. https://www.viasat.com/perspectives/corporate/2022/ka-sat-network-cyber-attack-overview/.

[43] Barenhorst F., Klein F., Barth S. (2023). Report 2022: Germany. IEA Wind TCP Annual Report 2022, Country Report; IEA Wind Technology Collaboration Programme. https://iea-wind.org/wp-content/uploads/2023/10/Germany_2022.pdf.

[44] Arsal M., Kamel T., Asad H., Khan A. (2026). A systematic review of cyber risk analysis approaches for wind power plants. Energies.

[45] Hink R.C.B., Beaver J.M., Buckner M.A., Morris T., Adhikari U., Pan S. (2014). Machine learning for power system disturbance and cyber-attack discrimination. Proceedings of the 7th International Symposium on Resilient Control Systems (ISRCS).

[46] Abukhousa E., Afroz S.S.F.S., Alsaeed F., Qwbaiban A., Meliopoulos A.P.S. (2025). Centralized Dynamic State Estimation Algorithm for Detecting and Distinguishing Faults and Cyber Attacks in Power Systems. Proceedings of the 2025 IEEE Power & Energy Society General Meeting (PESGM).

[47] Vaddi P.K., Diao X., Zhao Y., Smidts C. (2026). Dynamic probabilistic risk assessment and game theory for cyber security risk analysis in nuclear power plants. Reliab. Eng. Syst. Saf..

[48] Xue X., Shen D., Ding S.X., Zhao D. (2025). Dual Detection Framework for Faults and Integrity Attacks in Cyber-Physical Control Systems. arXiv.

[49] Ahsan M.S., Wang H., Motakatla V.R., Zhu M., Liu P. (2026). Differentiation Between Faults and Cyberattacks through Combined Analysis of Cyberspace Logs and Physical Measurements. arXiv.

[50] Das L., Gjorgiev B., Sansavini G. (2024). Uncertainty-aware deep learning for monitoring and fault diagnosis from synthetic data. Reliab. Eng. Syst. Saf..

[51] Fleming K.N., Silady F.A. (2002). A risk informed defense-in-depth framework for existing and advanced reactors. Reliab. Eng. Syst. Saf..

[52] Xing L., Distefano S. (2022). Reliability and performance of cyber-physical systems. Reliab. Eng. Syst. Saf..

[53] Yao P., Yang Q., Wang W. (2025). Dynamic cross-layer security risk assessment and mitigation for cyber-physical power systems. Reliab. Eng. Syst. Saf..

[54] Thapa M., Missoum S. (2022). Uncertainty quantification and global sensitivity analysis of composite wind turbine blades. Reliab. Eng. Syst. Saf..

[55] Zhu D., Huang X., Ding Z., Zhang W. (2024). Estimation of wind turbine responses with attention-based neural network incorporating environmental uncertainties. Reliab. Eng. Syst. Saf..

[56] Zhou T., Zhang L., Han T., Droguett E.L., Mosleh A., Chan F.T.S. (2023). An uncertainty-informed framework for trustworthy fault diagnosis in safety-critical applications. Reliab. Eng. Syst. Saf..

[57] Li H., Jiao J., Liu Z., Lin J., Zhang T., Liu H. (2025). Trustworthy Bayesian deep learning framework for uncertainty quantification and confidence calibration: Application in machinery fault diagnosis. Reliab. Eng. Syst. Saf..

[58] Page E.S. (1954). Continuous inspection schemes. Biometrika.

[59] Downs J.J., Vogel E.F. (1993). A plant-wide industrial process control problem. Comput. Chem. Eng..

[60] Gück C., Roelofs C.M.A., Faulstich S. (2024). CARE to Compare: A Real-World Benchmark Dataset for Early Fault Detection in Wind Turbine Data. Data.

[61] EDP–Energias de Portugal (2017). Wind Turbine SCADA Signals and Historical Failure Logbook (Open Data). EDP Open Data. https://www.edp.com/en/innovation/wind-farm-1-wind-turbine-scada-signals-2017.

[62] Clerc A., Lingkan E. (2026). Hill of Towie Wind Farm Open Dataset, Version 2.0.0. https://zenodo.org/records/20204946.

[63] Hadjidemetriou L., Asprou M., Ciornei I., Charalambous C., Tekki E. (2024). Dataset SUC1/S1–S4: Cyberattack Scenarios on DER Energy Management and Control. https://zenodo.org/records/12773981.

[64] Cibin N., Kabbara N., Presekal A., Semertzis I., Rajkumar V., Goyel H., Palensky P., Stefanov A. (2025). Cyber-Physical Power System Dataset for Cyber Security of Digital Substations. https://zenodo.org/records/15371179.

