# Supplementary material for: Identifiability-Aware, Cost-Aware Triage of Physical Faults and Measurement-Integrity Anomalies in Energy Cyber-Physical Systems
Source: Sensors (Basel). 2026 Sep 2;26(17):5585. doi: 10.3390/s26175585 (PMC13568006; doi:10.3390/s26175585)
Supplement: Supplementary file 1 [file sensors-26-05585-s001.zip › sensors-4492883-supplementary.pdf]

Supplementary Materials: Identifiability-Aware, Cost-Aware Triage of Physical Faults and Measurement-Integrity Anomalies in Energy Cyber-Physical Systems

Fuliang Ma [1], Yuzhen Dang [1], Yuanming Liu [2] and Tiezhuang Zhou [1,*]

Data and code availability. The reproducibility archive submitted with the main article contains the released analysis scripts, frozen result checkpoints, claims-to-code index, data cards, wind-farm simulator, and synthetic attack generator. Public dataset sources and licenses are identified in the main article and the data cards.

S1. Proofs and Extended Remarks for the Detectability Theory

This section collects the proofs of Propositions 1–4 of the main text, together with the extended remark on the correct instantiation of the contraction rate γ in the single-channel numerical validation.

S1.1. Proof of the SPE-only phase transition (main-text Proposition 1)

Proof. $\operatorname{rank}(R_{\mathcal{S}}) = \operatorname{rank}(\Pi^{\perp} E_{\mathcal{S}}) \leq \operatorname{rank}(\Pi^{\perp}) = n - \ell = d$. For $s \leq d$, a generic s-subset of columns of $\Pi^{\perp}$ (i.e. $\Pi^{\perp}$ acting on the s selected coordinate directions) is linearly independent, giving full column rank and $\sigma_{\min}(R_{\mathcal{S}}) > 0$; case 1 then follows from the covert-budget remark of the main text by letting $\|\tilde{a}\| \to \infty$. For $s > d$, the column count exceeds the maximal rank d, so $\dim\ker(R_{\mathcal{S}}) \geq s - d \geq 1$; any $0 \neq \tilde{a} \in \ker(R_{\mathcal{S}})$ yields $\Pi^{\perp} u = 0$ regardless of $\|\tilde{a}\|$, i.e. no SPE residual signature. □

S1.2. The deployed $T^2 \vee \mathrm{SPE}$ monitor has no phase transition (main-text Remark 1)

The deployed Stage 1 is an OR-combination: an alarm fires when *either* the SPE statistic *or* the T^2 statistic exceeds its calibrated limit. Write the combined normalized quadratic form on support $\mathcal{S}$ as $B_{\mathcal{S}} = E_{\mathcal{S}}^{\top} M E_{\mathcal{S}}$ with $M := P\,\operatorname{diag}(1/\lambda) P^{\top}/t_{\lim} + \Pi^{\perp}/Q_{\alpha}$ (main-text B-4 bound). The matrix M is positive definite on all of $\mathbb{R}^n$, by a complementary-kernel argument that requires no genericity: for any $x \in \mathbb{R}^n$, $x^{\top} M x = 0$ forces both $P^{\top} x = 0$ and $\Pi^{\perp} x = 0$; the second condition places x in $\operatorname{range}(P)$, the first makes x orthogonal to $\operatorname{range}(P)$, and these subspaces intersect only at the origin, so $x = 0$. (Neither summand is positive definite by itself: the first has rank $\ell < n$ and the second rank $d < n$, so an argument based on the rank of either term alone cannot establish $B_{\mathcal{S}} \succ 0$ once s exceeds that term's rank; it is the complementarity of their kernels that does.) Since the standardized coordinate embedding $E_{\mathcal{S}}$ has full column rank s for every support, $B_{\mathcal{S}} = E_{\mathcal{S}}^{\top} M E_{\mathcal{S}} \succ 0$ for every nonempty support $\mathcal{S}$ of every size, including $s > d$. Consequently $q(\tilde{a}) = \tilde{a}^{\top} B_{\mathcal{S}} \tilde{a} > 0$ for every unit $\tilde{a}$, so $\sigma_{\min}$ of the combined map never vanishes and *no support size admits an arbitrary-magnitude undetectable attack against the combined monitor*: the $s^{\star} = d + 1$ collapse of main-text Proposition 1 (SPE-only phase transition) is specific to the SPE channel alone. A null-space direction $\tilde{a} \in \ker(R_{\mathcal{S}})$ of that proposition's case 2 hides only the SPE component; it *always* leaves the T^2 component nonzero and quadratically amplified with magnitude (since $\tilde{a} \in \ker(R_{\mathcal{S}})$ means $\Pi^{\perp} E_{\mathcal{S}} \tilde{a} = 0$, the entire injection $E_{\mathcal{S}} \tilde{a} \neq 0$ lies in $\operatorname{range}(P)$, so its T^2 contribution scales with the squared magnitude), so the T^2 arm alone forces detection at large magnitude. The correct reading of the main-text covert-budget definition and the SPE-only phase transition is therefore not "the deployed system loses detectability past $s^{\star}$," but the narrower statement: the combined monitor loses detectability only if an attacker simultaneously evades *both* arms, and we do not claim any support size at which that joint evasion is guaranteed to succeed.

S1.3. Proof of the stealthy direction and innovation signature (main-text Proposition 2)

Proof. The first claim is the variational characterization of the smallest singular value: $\min_{\|\tilde{a}\|=1}\|R_{\mathcal{S}}\tilde{a}\| = \sigma_{\min}(R_{\mathcal{S}})$, attained at the corresponding right-singular vector. For the second, let the normal dynamics admit a stable one-step predictor $\hat{z}_{k|k-1} = A\hat{z}_{k-1|k-1}$, $\hat{z}_{k|k} = \hat{z}_{k|k-1} + K\nu_k$, with innovation $\nu_k = z_k - \hat{z}_{k|k-1}$ and Schur closed-loop error map $A(I-K)$ (here $C = I$ in the measurement coordinates, so $KC = K$). Adding a *constant* standardized injection u to the measured stream, $z_k \mapsto z_k + u$, and writing $m_k := \mathbb{E}[\hat{z}_{k|k}]$ for the mean of the filtered estimate (the true state has mean zero under stable, zero-mean normal dynamics), the mean recursion is

$$m_k = Am_{k-1} + K\nu_k, \qquad \nu_k = u - Am_{k-1},$$

so that $m_k = (I-K)Am_{k-1} + Ku$. The recursion matrix $(I-K)A$ shares its spectrum with $A(I-K)$ (for square M, N the products MN and NM have the same eigenvalues), hence is Schur by assumption, and the recursion has the unique fixed point

$$m_\infty = \big(I - (I-K)A\big)^{-1}Ku.$$

The stationary innovation mean is then

$$\begin{aligned}\mathbb{E}[\nu_\infty] &= u - Am_\infty \\ &= u - A\big(I-(I-K)A\big)^{-1}Ku \\ &= u - \big(I - A(I-K)\big)^{-1}AKu \\ &= \big(I - A(I-K)\big)^{-1}(I-A)\,u,\end{aligned}$$

where the third equality is the push-through identity $A\big(I-(I-K)A\big)^{-1} = \big(I - A(I-K)\big)^{-1}A$ and the last follows by multiplying out the bracketed expression, which reduces to $\big(I-A(I-K)\big)^{-1}(I-A)$. Hence $\mathbb{E}[\nu_\infty] = (I-\Phi)u$ with

$$\Phi \;:=\; \big(I - A(I-K)\big)^{-1}AK,$$

the predictor's *DC gain* from a constant measurement offset to the one-step prediction (well defined because $A(I-K)$ is Schur, so $I - A(I-K)$ is invertible). (Numerical check: for the scalar case $A = K = 0.5$, direct iteration of the recursion gives $\Phi = 1/3$ and $\mathbb{E}[\nu_\infty] = (2/3)u$, matching the closed form exactly; a random 3×3 Schur-stable A with diagonal gain K confirms $I - \Phi = (I - A(I-K))^{-1}(I-A)$ to numerical precision against the simulated steady state.) Now, $I-\Phi = (I-A(I-K))^{-1}(I-A)$ is the product of an invertible matrix and $(I-A)$, so $\ker(I-\Phi) = \ker(I-A)$ exactly. Because A is Schur-stable, 1 is not an eigenvalue of A, so $I-A$ is invertible and $\ker(I-A) = \{0\}$. Hence $\mathbb{E}[\nu_\infty] = 0$ iff $u = 0$: unlike the SPE-silent set $\ker(R_{\mathcal{S}})$, whose dimension is $\geq s-d > 0$ once $s > d$, the innovation-silent set for a constant offset is trivial for *every* support size and every Schur-stable A; there is no non-generic eigenvalue condition to hedge on. Consequently, whenever $s > d$ and a nonzero $\tilde{a} \in \ker(R_{\mathcal{S}})$ exists (main-text Proposition 1, case 2), the corresponding constant injection is exactly SPE-silent but strictly innovation-detected: the innovation-silent and SPE-silent sets intersect only at $u = 0$. □

S1.4. Proof of measurement-only causal non-identifiability (main-text Proposition 3)

Let A be the event that a deterministic or randomized rule outputs atk. Equality of the conditional laws implies

$$q := \Pr(A \mid C = \text{sen}) = \Pr(A \mid C = \text{atk}).$$

Attack recall is q, whereas sensor-fault specificity is $1 - q$. Consequently their mean, the balanced accuracy, equals $(q + 1 - q)/2 = 1/2$ for every rule. With equal class priors the ordinary accuracy is also $1/2$, so the minimum achievable classification error is $1/2$.

For a real-valued score $S = s(Y)$, equality of the conditional laws of Y implies equality of the conditional laws of S. If S_{atk} and S_{sen} are independent draws from that common distribution, exchangeability gives

$$\Pr(S_{\text{atk}} > S_{\text{sen}}) = \Pr(S_{\text{sen}} > S_{\text{atk}}).$$

Adding half of the tie probability to either ordering yields AUC $= 1/2$. Finally, if two finite-sample inputs are exact copies, every deterministic transformation and fitted prediction function returns the same value for both. Randomization cannot improve expected performance because the preceding argument already permits randomized rules.

S1.5. Proof of the UUB guarantee (main-text Proposition 4)

A naive version of this argument would absorb the clean-channel saturation residual into the noise constant; that step is circular (the residual is state-dependent), and it omits the constant relating the Lyapunov sublevel set to the error norm. The proof below closes both gaps: part (a) is a coarser bound that holds *unconditionally*; part (b) recovers the sharper main-text bound under an explicit, checkable saturation-margin condition, with the conditioning factor $\sqrt{\lambda_{\max}(S)/\lambda_{\min}(S)}$ now stated.

Proof. Partition the measured innovation by attacked/clean support, $\nu_k = [\nu_{k,\mathcal{S}}; \nu_{k,\mathcal{S}^c}]$, and write the injected corruption on the attacked channels as a_k (supported on $\mathcal{S}$, otherwise arbitrary and possibly unbounded). The measured innovation on $\mathcal{S}$ is $\tilde{\nu}_{k,\mathcal{S}} = \nu_{k,\mathcal{S}} + a_k$, while $\nu_{k,\mathcal{S}^c}$ is clean. The estimator applies the gain to the *saturated* measured innovation. Keeping the attacked-channel correction *whole* (splitting off its clean part would re-introduce the state-dependent quantity $\nu_{k,\mathcal{S}} = \Pi_{\mathcal{S}} e_k$ into the disturbance and make the bound circular), the error recursion splits as

$$e_{k+1} = \bar{A}\, e_k + w_k \; - \; L_{:,\mathcal{S}}\, \text{sat}_\rho(\tilde{\nu}_{k,\mathcal{S}}) \; - \; L_{:,\mathcal{S}^c} \underbrace{\left(\text{sat}_\rho(\nu_{k,\mathcal{S}^c}) - \nu_{k,\mathcal{S}^c}\right)}_{=:\delta_k}, \tag{S1}$$

where $\bar{A} = A - L_{:,\mathcal{S}^c}\Pi_{\mathcal{S}^c}$ collects the clean-channel feedback. The attacked-channel correction $L_{:,\mathcal{S}}\, \text{sat}_\rho(\tilde{\nu}_{k,\mathcal{S}})$ is the *cross-channel corruption pathway* that a single-channel analysis would omit: it is the component that $L_{:,\mathcal{S}}$ propagates into *all* state estimates, including those of the clean channels $\mathcal{S}^c$ through the off-diagonal block $L_{\mathcal{S}^c,\mathcal{S}}$. Because sat_ρ maps into the coordinate-wise ball of radius ρ, $\|\text{sat}_\rho(\tilde{\nu}_{k,\mathcal{S}})\| \leq \rho\sqrt{s}$ at every step, so the attack contributes at most $\|L_{:,\mathcal{S}}\|\,\rho\sqrt{s}$ regardless of $\|a_k\|$, with no recourse to any a-priori bound on the attacked-channel innovation itself.

Weighted-norm contraction. For $S \succ 0$ define $\|x\|_S = \sqrt{x^\top S x}$, so $\sqrt{\lambda_{\min}(S)}\,\|x\| \leq \|x\|_S \leq \sqrt{\lambda_{\max}(S)}\,\|x\|$. A Stein pair $M^\top S M - S \preceq -\epsilon I$ gives, for every x, $\|Mx\|_S^2 \leq \|x\|_S^2 - \epsilon\|x\|^2 \leq \left(1 - \epsilon/\lambda_{\max}(S)\right)\|x\|_S^2$, i.e. $\|Mx\|_S \leq \gamma\|x\|_S$ with $\gamma^2 = 1 - \epsilon/\lambda_{\max}(S) <$

1. For any recursion $e_{k+1} = Me_k + d_k$ with $\|d_k\| \le D$ this yields $\|e_{k+1}\|_S \le \gamma\|e_k\|_S + \sqrt{\lambda_{\max}(S)}\,D$, hence

$$\limsup_{k\to\infty}\|e_k\| \;\le\; \frac{1}{\sqrt{\lambda_{\min}(S)}}\;\limsup_{k\to\infty}\|e_k\|_S \;\le\; \sqrt{\tfrac{\lambda_{\max}(S)}{\lambda_{\min}(S)}}\;\frac{D}{1-\gamma}, \tag{S2}$$

and moreover every sublevel set $\{\|e\|_S \le r\}$ with $r \ge \sqrt{\lambda_{\max}(S)}D/(1-\gamma)$ is forward-invariant.

(a) Unconditional coarse bound. Group the *entire* correction as a disturbance: $e_{k+1} = Ae_k + w_k - L\,\mathrm{sat}_\rho(\tilde{\nu}_k)$ with $\|L\,\mathrm{sat}_\rho(\tilde{\nu}_k)\| \le \|L\|\rho\sqrt{n}$. Since A is Schur by standing assumption it admits a Stein pair (S_A, ϵ_A), and (S2) applies with $M = A$, $D = \bar{w} + \|L\|\rho\sqrt{n}$, giving UUB unconditionally (for *every* ρ and every support) at the coarser rate γ_A and dimension factor $\sqrt{n}$. Denote the resulting ultimate bound R_1.

(b) Sharper bound under the saturation-margin condition. Now use (S1). Componentwise $|\delta_k| \le \max(0, |\nu_{k,\mathcal{S}^c}| - \rho)$, so $\delta_k = 0$ whenever $\|\nu_{k,\mathcal{S}^c}\|_\infty \le \rho$. The clean innovation obeys $\|\nu_{k,\mathcal{S}^c}\|_\infty \le \|\Pi_{\mathcal{S}^c} e_k\| + \bar{w}_\nu \le \|e_k\| + \bar{w}_\nu$, where $\bar{w}_\nu$ bounds the noise contribution to the innovation. Let $\kappa := \sqrt{\lambda_{\max}(S)/\lambda_{\min}(S)}\,\|L_{:,\mathcal{S}}\|\sqrt{s}\,/\,(1-\gamma)$ and impose the **saturation-margin (small-gain) condition**

$$\kappa < 1 \qquad \text{and} \qquad \rho\,(1-\kappa) \;\ge\; \sqrt{\tfrac{\lambda_{\max}(S)}{\lambda_{\min}(S)}}\;\frac{\bar{w}}{1-\gamma} \;+\; \bar{w}_\nu. \tag{S3}$$

Define $R_2 := \sqrt{\lambda_{\max}(S)/\lambda_{\min}(S)}\,(\bar{w} + \|L_{:,\mathcal{S}}\|\rho\sqrt{s})/(1-\gamma)$; condition (S3) is exactly $R_2 + \bar{w}_\nu \le \rho$. Work on the S-norm sublevel set $\Omega := \{\|e\|_S \le \sqrt{\lambda_{\min}(S)}\,(\rho - \bar{w}_\nu)\}$. (In Euclidean terms Ω contains the ball $\|e\| \le (\rho - \bar{w}_\nu)\sqrt{\lambda_{\min}(S)/\lambda_{\max}(S)}$; for ill-conditioned S it need *not* contain all of $\{\|e\| \le R_2\}$: the two sets differ by the conditioning factor, which is why every containment below is stated in the S-norm.) On Ω, $\|e\| \le \|e\|_S/\sqrt{\lambda_{\min}(S)} \le \rho - \bar{w}_\nu$, so the clean innovations satisfy $\|\nu_{\mathcal{S}^c}\|_\infty \le \rho$, hence $\delta_k = 0$ and (S1) reduces to $e_{k+1} = \bar{A}e_k + d_k$ with $\|d_k\| \le \bar{w} + \|L_{:,\mathcal{S}}\|\rho\sqrt{s} =: D_2$. Condition (S3) is equivalent to $\sqrt{\lambda_{\min}(S)}(\rho - \bar{w}_\nu) \ge \sqrt{\lambda_{\max}(S)}\,D_2/(1-\gamma)$, so by the invariance clause of (S2) applied to $M = \bar{A}$, Ω itself is forward-invariant and the reduction $\delta_k = 0$ persists along every trajectory starting in Ω; those trajectories converge to the ultimate bound R_2, the main-text bound, now carrying the explicit conditioning factor. If additionally $\rho \ge \sqrt{\lambda_{\max}(S)/\lambda_{\min}(S)}\,R_1 + \bar{w}_\nu$ (the coarse Euclidean ball of part (a) must fit inside Ω, which costs another conditioning factor), part (a) drives *every* trajectory into Ω in finite time, making R_2 global; otherwise R_2 holds for initial errors in Ω and R_1 holds globally. When $\mathcal{S}^c = \emptyset$ there is no clean channel, δ_k does not exist, condition (S3) is vacuous, and R_2 holds globally with $\bar{A} = A$; in the scalar single-channel validation S is scalar, the conditioning factor is 1, and the published closed form is recovered unchanged. The clean-channel leakage is explicit in the recursion: if $L_{\mathcal{S}^c,\mathcal{S}} = 0$ the attacked innovation cannot corrupt the $\mathcal{S}^c$ estimates and the cross term drops out. □

Scope remark. The Stein condition, and hence the guarantee, is stated *per attacked support* $\mathcal{S}$: uniformity over a family of admissible supports requires verifying the condition for each member ($\binom{n}{s}$ in general). A gain L that is block-diagonal with respect to a partition of channels reduces this to one condition per block, at the price of ignoring cross-block correlations; we use the per-support statement and flag the design trade-off rather than claim uniformity.

S1.6. Multichannel validation of the UUB bound

The single-channel validation of the main text (experiments section) exercises only the scalar case; the cross-channel leakage path $L_{\mathcal{S}^c,\mathcal{S}}$ of the bound (main-text Eq. for

Table S1. Sensitivity of the scalar resilient-estimator validation to the innovation-saturation radius ρ.

ρ	UUB bound	Steady-state error	Detection delay	Miss fraction
1.0	9.681	1.673	17.43	0.00
1.5	10.451	2.341	11.13	0.00
2.0	11.221	3.083	9.20	0.00
3.0	12.761	4.617	7.77	0.00
4.0	14.301	6.155	7.50	0.00
6.0	17.381	9.232	7.43	0.00

Notes: $A = 0.610$, $L = 0.6$, $\bar{w} = 3.172$, $s = 1$, and $\gamma = |A|$. Steady-state values average 30 repetitions after a sustained bias of 20 noise-scale units. Detection uses a four-unit sensor step after 200 pre-onset samples and the one-sided recursion $g_k = \max(0, g_{k-1} + |\,\mathrm{sat}_\rho(\nu_k)| - 0.25)$ with alarm $g_k > 12$; each trial permits 1500 post-onset samples. Noise is Gaussian truncated at $\bar{w}$. The table is generated directly by `analysis/06_theory_experiments.py`.

$\limsup \|e_k\|$) requires $n > 1$ to appear. We therefore ran a *multichannel* validation ($n = 4$; attack support $s \in \{1, 2, 3\}$; cross-channel gain couplings $L_{\mathrm{off}} \in \{0, 0.12, 0.30\}$; 21 configurations in total). The ultimate error bound holds in all 21 configurations, with the steady-state error magnitude-independent throughout (a 1×–128× injection sweep per configuration). The $\sqrt{s}$ scaling of the attack-driven term is exact under a block-diagonal gain and modulated by $\|L_{:,\mathcal{S}}\|$ otherwise, exactly as the closed form predicts. One further observation is disclosed for auditability: the saturation-margin (small-gain) condition of the sharp bound *can* be violated in practice—clean-channel innovations exceeded ρ in 10 of the 21 configurations, up to 48.5% of steps under strong coupling and small ρ. Although the bound was never breached empirically in those runs, its formal guarantee there rests on the coarser unconditional bound of the UUB proof above rather than the sharp one.

S1.7. Instantiation of γ in the single-channel validation

Remark S1 (Correct instantiation of γ (*proved*))**.** *The UUB bound defines γ as the Stein-condition contraction rate of $\bar{A} = A - L_{:,\mathcal{S}^c}\Pi_{\mathcal{S}^c}$, the* clean-channel *error dynamics. When the numerical validation of the main text (the UUB-validation figure in main-text §6 and Table S1) uses a single scalar channel that is* entirely *under attack, i.e. $\mathcal{S}$ is the whole (one-dimensional) state and $\mathcal{S}^c = \emptyset$, the clean-channel term vanishes identically, $\bar{A} = A$, so the contraction rate that must appear in the denominator of the bound is $\gamma = |A|$ (the bare, uncorrected dynamics),* not *$|A - L|$: with $\mathcal{S}^c = \emptyset$ there is no clean channel whose feedback loop $A - L$ could contract, since the gain L is applied to the saturated (attacked) innovation and enters the recursion only through the numerator term $L_{:,\mathcal{S}}\rho\sqrt{s}$, exactly as* (S1) *shows. Using $\gamma = |A - L|$ in this single-channel case would understate the bound whenever $|A - L| < |A|$ (as for the identified constants $A \approx 0.610$, $L = 0.6$, where $|A - L| \approx 0.010 \ll |A|$). With $\gamma = |A|$, the closed form $(\bar{w} + L\rho\sqrt{s})/(1 - \gamma)$ strictly exceeds the empirical steady-state error at every ρ tested (Table S1).*

S2. Strict Learned-Model Comparison

The primary comparison changes the Stage-2 representation and classifier without changing the fixed CVA gate, commissioning centering, outer folds, or run aggregation. The physical model is a 500-tree random forest with minimum leaf size 2 and balanced class weights. The learned comparator is XGBoost (300 trees, maximum depth 4, learning rate 0.05, row and column subsampling 0.9). Both receive identical detected runs in five stratified complete-run outer folds.

Raw statistics concatenate the mean, standard deviation, minimum, and maximum of every standardized channel (208 dimensions for TEP and 56 for the wind farm). Both representations are expressed as changes from the same episode-specific commissioning median. The raw model's higher AUC establishes the clean-data accuracy benchmark. The

Table S2. Conditional outer-fold run-level AUC under the locked protocol.

Representation and model	TEP	Wind farm
Nine physical-consistency features + RF	0.873	0.895
Raw window statistics + XGBoost	0.949	0.958

physical model's contribution is a compact, inspectable evidence chain; the data do not justify describing it as the most accurate classifier.

For protocol transparency, the archive also contains the multi-model and shift-sensitivity scripts. Their repeated-split outputs are secondary sensitivity analyses and are not used for the strict headline estimates.

S3. Strict Protocol Disaggregated by Mode

Disaggregation uses the same five outer folds and the same one-score-per-run rule as the main result. No threshold is refitted by family or fault mode.

TEP provides one run per standard IDV mode, so mode-level classification recall is not estimated. The alarm-window fractions reveal the principal gate limitations: IDV3 is 0.103, IDV9 is 0.026, and IDV15 is 0.308. At the attack-family level, TEP bias is the main Stage-2 error source, whereas the wind-farm gate is weakest for slowly developing pitch and aerodynamic faults. These strata should guide additional run generation rather than support causal claims about mechanism difficulty.

S4. Calibration, Cost, and Sensitivity Protocols

S4.1. Nested Run-Level Calibration

For every outer fold, one stratified inner complete-run split supplies calibration runs. A logistic map is fitted to the logit of run-averaged random-forest probabilities and then applied once to each outer test run. The process produces exactly 68 TEP and 75 wind-farm test probabilities; no run appears in its own classifier or calibrator fit.

The mixed direction across metrics is the reason raw scores are primary. Although AUC is unchanged by a monotone map, classification and cost can change because the operating threshold is fixed.

S4.2. Exact Cost Accounting

For each episode, the action is evaluated directly against the normalized matrix $\begin{bmatrix} 1 & 30 \\ 50 & 5 \end{bmatrix}$. Stage-1 misses receive the off-diagonal consequence because the pipeline cannot route them correctly. The same miss charge is applied to the blanket comparators. Table S6 is generated from `results/primary_analysis.json`.

S4.3. Secondary Sensitivity Analyses

The reproducibility package includes scripts for broader cost grids, sensor noise perturbations, drift, cross-checking, and reject options. Those outputs use a repeated grouped-split protocol distinct from the locked five-fold analysis. They are included for transparency and hypothesis generation, but are excluded from the main claims because protocol changes would otherwise be confounded with model changes. A future robustness study should prespecify perturbations, refit neither thresholds nor deferral bands on test data, and evaluate complete held-out runs.

Table S3. Attack-family coverage and conditional recall at threshold 0.5. Each family contains 12 runs.

	TEP		Wind farm	
Family	Coverage	Recall	Coverage	Recall
Bias	1.000	0.667	1.000	0.833
Denial of service	1.000	1.000	1.000	0.833
Replay	1.000	1.000	1.000	1.000
Stealthy	0.917	1.000	1.000	1.000

Table S4. Wind-farm fault-mode Stage-1 coverage, alarm-window fraction, and end-to-end recall. Each mode contains five runs; values are therefore descriptive.

Mode	Coverage	Alarm-window fraction	End-to-end recall
Gearbox-bearing degradation	1.000	0.856	1.000
Generator overheating	1.000	0.277	0.400
Pitch-system fault	0.600	0.062	0.400
Drivetrain imbalance	1.000	0.241	1.000
Converter-cooling fault	1.000	0.821	0.800
Aerodynamic degradation	0.800	0.077	0.400

Table S5. Strict outer-fold calibration diagnostics. Lower is better for Brier score, negative log-likelihood (NLL), and ECE.

Dataset	Score	Brier	NLL	ECE	Bal. acc.
TEP	Raw	0.1204	0.3817	0.0819	0.8146
	Platt	0.1163	0.3766	0.0966	0.7751
Wind farm	Raw	0.1199	0.3860	0.1090	0.8287
	Platt	0.1222	0.3778	0.1033	0.8021

Table S6. Exact episode-level EMC, including all detector misses.

Policy	TEP	Wind farm
Always maintenance after alarm	35.087	32.269
Always isolation after alarm	13.261	14.615
Physical score, raw	10.826	10.397
Physical score, Platt	8.638	11.141

S5. Auxiliary Real-Data Evidence: EDP, Hill of Towie, and ELECTRON/KIOS SUC1

This section gives the full protocols and results for the three secondary real-data checks summarized in the main-text real-data validation section. None is a paired real-fault/real-attack attribution test; they triangulate interpretability, false-alarm-rate robustness, and wind-specific attack provenance, respectively.

EDP (named-signal interpretability). On the EDP open wind-turbine SCADA dataset [1], whose signals are natively named and whose failure logbook lists 16 component failures (generator 6, generator bearing 4, gearbox 2, hydraulic 2, transformer 2), the dominant residual channel recovered for each synthetic attack family is physically coherent: bias→generator speed, replay→gearbox-oil temperature, DoS→rotor speed, stealthy→converter temperature. We read this as *illustrative* interpretability evidence on named signals rather than a systematic family-to-channel mapping, since the recovered channel depends on the sampled attack supports. The dataset's failure logbook is cited for context and does not enter any computation here. It complements CARE's larger but partly anonymized channel set.

Hill of Towie (real-field FAR robustness). On three years of real field SCADA from a 21-turbine farm [2] (2.5×10^6 ten-minute records), we calibrated the control limits once on the base year and measured the realized alarm rate in each subsequent year (main-text Figure, real-data validation panel (c)). The realized alarm rate stays within roughly 1.3–4.2% against the 3% calibration target across all detectors and years, quantifying threshold robustness to real operating-regime drift. Hill of Towie carries no curated fault labels, so the measured quantity is the *total* alarm rate (an upper bound on the false-alarm rate, since any genuine anomalies in those years inflate it). The dataset is used only as a threshold-stability check, not a discrimination benchmark.

ELECTRON/KIOS SUC1 (wind-specific attack provenance). To strengthen the attack side of the evidence, we audit the public ELECTRON/KIOS SUC1 sandbox data [3]: a wind-specific cyber-physical sandbox driven by 30-s field measurements from an operating 10.8-MW wind farm. Its scenarios implement MITM false-data injections on the wind-farm power measurement (S1), a battery-storage measurement (S2) and set-point (S3), and an MITM/DoS *actually executed* on an isolated Modbus-TCP network, with packet capture (S4). With the timestamp, error flag, and all ground-truth channels excluded, the paper's Stage-1 discipline (a normal-only calibrated PCA T^2/SPE monitor at a 3% held-out-normal limit) detects 3 of the 4 attack events under the $\geq$ 3-consecutive-sample rule. The per-scenario attack-sample counts are small and we report them with Wilson 95% intervals rather than bare ratios: S2 21/22 (0.955, $[0.782, 0.992]$), S3 16/22 (0.727, $[0.518, 0.868]$), S4 18/18 (1.000, $[0.824, 1.000]$), while the S1 injection remains a documented miss (1/22, 0.045, $[0.008, 0.218]$; no event). S1's realized false-alarm rate on non-attack rows is 6.8% (15/220, $[0.042, 0.109]$), above the 3% target, so the S1 scenario fails on both axes and we count it plainly as a miss. This is attack-side evidence on wind-specific, field-driven data with a real executed network attack: not an intrusion observed on the operating farm, and not a paired real-fault/real-attack attribution test (`run_electron_suc1_audit.py` in the released kit).

S6. Benign-Sensor-Fault and Identifiability Stress Test

S6.1. Declared data objects and generation

The extension contains two explicitly different synthetic data objects. *Exact matched controls* are causal relabels of existing simulated attack runs: calibration bias is paired with additive bias, a stale acquisition buffer with replay, and stuck-at output with denial-of-service freezing. Within each pair the full received array is copied bit for bit. There are 12

pairs per family and 36 pairs per domain. The paired records share an equivalence-group identifier and outer fold.

Distinctive mechanism probes are generated only for the simulated wind-farm case. Six independent healthy simulator seeds are used for each of calibration drift, noise inflation, stuck-at output, intermittent sample hold, quantization, and isolated spikes, giving 36 runs. Each run has the same 160-sample commissioning boundary as the primary protocol. The sensor fault is injected only after that boundary. These are controlled synthetic mechanism probes, not empirical estimates of field fault prevalence, severity, or frequency.

S6.2. Detection and three-class accounting

Table S7 reports the complete Stage-1 accounting. The conditional three-class confusion matrices in Figure S1 then use one complete-run median feature vector per detected episode. A Stage-1 miss remains an error in the end-to-end macro recall reported in the main text.

Table S7. Stage-1 run detection in the sensor-fault extension.

Domain	Process fault	Sensor fault	Attack
TEP	21/21	36/36	47/48
Wind-farm simulation	27/30	72/72	48/48

Notes: Sensor-fault counts include 36 exact matched causal relabels in each domain and, for the wind-farm simulation only, 36 distinctive mechanism probes.

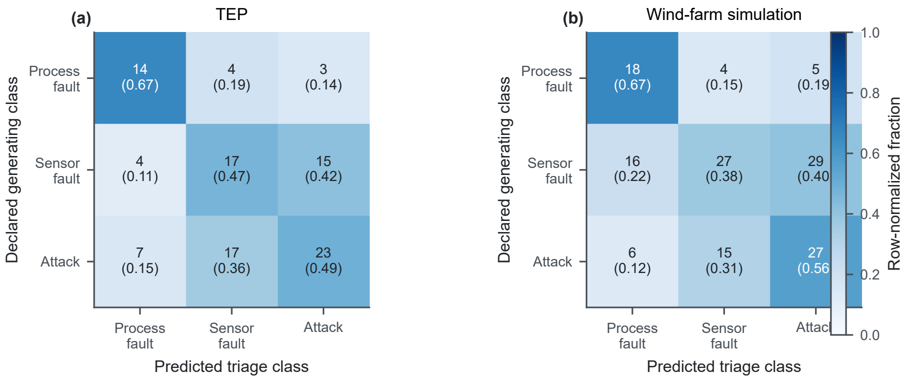

Figure S1. Out-of-fold three-class confusion matrices for the nine physical-consistency features. Cell text gives the run count and row-normalized fraction. Rows are declared generating classes, not field-verified causes.

S6.3. Selective cause decisions

The cause branch is evaluated only among the pooled measurement-integrity runs. A symmetric threshold t releases a sensor-fault decision when $p_{\text{attack}} \leq 1 - t$, releases an attack decision when $p_{\text{attack}} \geq t$, and otherwise returns review. Table S8 gives the prespecified $t = 0.75$ operating point; Figure S2 reports the full frozen sweep. Accuracy is conditional on a released decision and must always be read together with coverage.

Table S8. Selective sensor-fault/attack cause branch at threshold $t = 0.75$.

Domain	Model	Decision coverage	Covered accuracy	Matched-pair review rate
TEP	Physical features	0.024	0.500	0.972
TEP	Raw XGBoost	0.265	0.545	0.722
Wind-farm simulation	Physical features	0.117	0.643	0.861
Wind-farm simulation	Raw XGBoost	0.450	0.741	0.667

Notes: Coverage and covered accuracy concern only detected measurement-integrity runs. The threshold was declared in the configuration, not selected from these test-fold results. A high review rate is expected for exact observational equivalents.

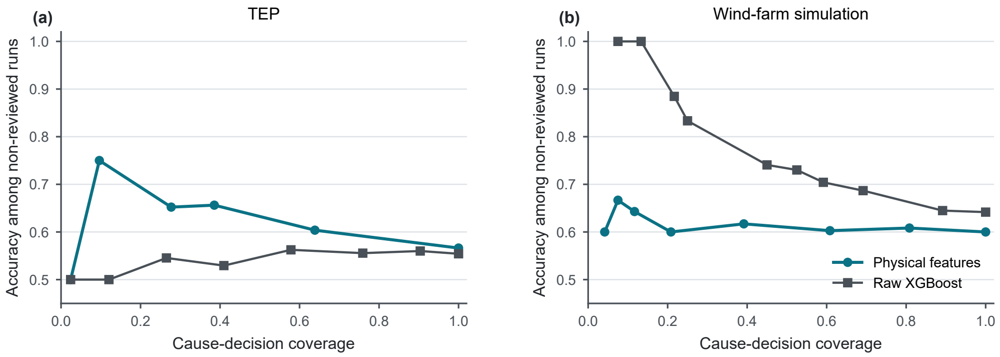

Figure S2. Coverage–accuracy trade-off for selective cause decisions. Thresholds run from 0.50 to 0.95 in increments of 0.05. Points with no released decisions have undefined accuracy and are omitted. This curve does not validate a deployable threshold because the causes are synthetic and the number of independent groups is limited.

S6.4. Leakage and interpretation audit

The mechanical audit verifies, for every exact pair, identical physical and raw vectors, a common multiclass fold, a common cause-branch fold, and valid probability normalization. It finds 36 equivalence groups per domain and no identity, fold, probability, or group-leakage errors. The maximum within-pair physical-model probability difference is 2.22×10^{-16} on TEP and zero on the wind-farm simulation; matched cause AUC is 0.500 for both models in both domains.

The result identifies the safe evidential boundary. Distinctive synthetic mechanisms can be learned because their measurement laws differ, but a benign/malicious cause is not recoverable when its received trajectory is the same. A field cause branch therefore requires non-equivalent side information: sensor self-test or quality flags, independent redundant measurements, maintenance and calibration records, authenticated network events, or forensic logs. No such signals are present in the current public wind benchmarks, and no field sensor-fault or cyber-causation performance is claimed.

References

1. EDP–Energias de Portugal. Wind Turbine SCADA Signals and Historical Failure Logbook (Open Data). EDP Open Data, 2017. Available online: https://www.edp.com/en/innovation/wind-farm-1-wind-turbine-scada-signals-2017 (accessed on 2 September 2026).
2. Clerc, A.; Lingkan, E. Hill of Towie Wind Farm Open Dataset, Version 2.0.0. 2026. Available online: https://doi.org/10.5281/zenodo.20204946 (accessed on 2 September 2026).
3. Hadjidemetriou, L.; Asprou, M.; Ciornei, I.; Charalambous, C.; Tekki, E. Dataset SUC1/S1–S4: Cyberattack Scenarios on DER Energy Management and Control. 2024. Available online: https://doi.org/10.5281/zenodo.12773981 (accessed on 2 September 2026).
